# Which exercise intervention is most promising for Parkinson's balance? A network meta-analysis

**DOI:** 10.3389/fnagi.2026.1879017

**Published:** 2026-07-15

**Authors:** Ying Li, Meiling Zhao, Zhaojun Luo, Yanxia Tang

**Affiliations:** 1 College of Sports Science, Jishou University, Jishou, Hunan, China; 2 School of Nursing, Qilu Medical University, Zibo, Shandong, China; 3 Department of Physical Education, Shenzhen Foreign Languages School (Guangming Campus), Shenzhen, Guangdong, China; 4 Normal College, Jishou University, Jishou, Hunan, China

**Keywords:** balance, dance exercise, meta-analysis, network meta-analysis, parkinson's disease

## Abstract

**Objective:**

Parkinson's disease (PD) is a chronic, progressive, and disabling neurodegenerative disorder. While bradykinesia, resting tremor, and rigidity are the cardinal motor symptoms, balance impairments and postural instability frequently emerge during disease progression, substantially increasing the risk of falls and diminishing quality of life. Although exercise is a well-established, effective non-pharmacological intervention for improving balance and motor function in individuals with PD, the comparative efficacy of different exercise modalities remains unclear. Therefore, this network meta-analysis aims to evaluate and compare the effects of various exercise therapies on balance outcomes in patients with PD, providing evidence-based guidance for optimizing exercise prescriptions.

**Method:**

We searched databases such as PubMed, EMBASE, Cochrane Library, Web of Science, and CNKI from establishing the database until August 2025. We screened literature based on inclusion and exclusion criteria and compared the effects of 30 different exercise interventions on balance ability in people with PD.

**Results:**

Our study included a total of 135 articles and 5948 People with PD. Our study found that Dance exercise (DE) is more effective than Control group (CON) (MD, 6.25; 95% CI, 2.81 to 9.69) and Traditional Rehabilitation (TR) (MD, 7.56; 95% CI, 3.89 to 11.23) in improving Berg Balance Scale (BBS) in Parkinson's disease patients. In terms of reducing the Timed Up and Go Test (TUG) score of people with PD, Biofeedback Balance and Gait Training (BGT) is superior to CON (MD, −10.47; 95% CI, −20.13 to −0.81) and TR (MD, −10.60; 95% CI, −20.05 to −1.15), Stretch exercise (SE) (MD, −14.30; 95% CI, −26.75 to −1.85), Virtual reality balance training (VRB) (MD, −12.85; 95% CI, −23.40 to −2.29) and Yoga (YG) (MD, −13.29; 95% CI, −23.97 to −2.61). Pilates exercise (PE) (MD, −13.47; 95% CI, −25.98 to −0.96).is superior to CON in improving Unified Parkinson's disease rating scale III (UPDRS-III) scores. Qigong (QG) is superior to TR (MD, −6.24; 95% CI, −10.87 to −1.60) and Resistance training (RT) (MD, −8.21; 95% CI, −16.10 to −0.33) and CON (MD, −8.89; 95% CI, −14.35 to −3.42) in improving UPDRS-III.

**Conclusion:**

Compared with the CON and TR groups, DE (as measured by the BBS) and BGT (as measured by the TUG) demonstrated superior efficacy in improving balance function in patients with PD. PE and QG showed greater benefits than CON in improving global motor symptoms, as assessed by the UPDRS-III. These findings may provide evidence-based guidance for clinicians, rehabilitation professionals, and patients when selecting exercise interventions tailored to specific therapeutic goals in PD. Nevertheless, the mechanisms underlying these modality-specific effects remain unclear and warrant further investigation. In addition, the observed superiority of BGT for TUG outcomes should be interpreted with caution, as this finding was derived from a single study and requires confirmation in future high-quality randomized controlled trials.

## 1 Introduction

Parkinson's disease (PD) is a chronic, progressive, and disabling neurodegenerative disorder that primarily affects older adults. Approximately 1%−5% of individuals aged 65 years and above are estimated to be living with this condition (Rocha et al., 2018). Moreover, the global burden of PD is expected to increase substantially, with the number of affected individuals projected to rise from 6.2 million in 2015 to nearly 12 million by 2040 (Dorsey and Bloem, 2018). The disease is characterized by cardinal motor symptoms, including bradykinesia, resting tremor, and rigidity, all of which can negatively affect postural control and balance (Jankovic, 2008). Balance refers to the ability to maintain control of body mass through the integration of sensory, musculoskeletal, and central nervous system functions in response to both internal and external environmental changes (Park et al., 2015). As a fundamental component of postural control and motor coordination, balance is essential for maintaining stability during both static and dynamic activities (Wallace et al., 2009). Balance disorders can make it difficult for patients to perform daily activities (Jacobs et al., 2009), reduce the perception of balance, and ultimately increase the risk of falls (Adkin et al., 2003). The annual incidence of falls in people with PD ranges from 35% to 90%, with mild falls causing bruising, soft tissue and bone injury, and severe ones causing fractures (Cummings and Eastell, 2016). In addition, falls may induce a fear of falling in people with PD. This fear can lead patients to intentionally reduce their participation in daily activities and social interactions. Consequently, reduced activity levels may contribute to a series of secondary complications, resulting in deterioration of overall health and an increased risk of rehospitalization. Therefore, early assessment and intervention targeting balance function are essential for people with Parkinson's disease (PwPD).

At present, there remains no cure for PD. While pharmacological interventions can alleviate both motor and non-motor symptoms, long-term pharmacotherapy demonstrates limited efficacy in improving motor dysfunction and does not halt disease progression (Grabli et al., 2012). Furthermore, the complexity of medication regimens can impose a substantial treatment burden on patients, contributing to poor adherence, including missed doses, incorrect dosing, and self-adjusted medication (Bakker et al., 2022). These behaviors may consequently worsen Parkinson's symptoms and increase the risk of drug withdrawal syndromes, ultimately diminishing patients' quality of life (Mucksavage and Kim, 2020). Therefore, effective non-pharmacological interventions are increasingly recognized as important complements to pharmacological treatment in the management of PD. Among these interventions, exercise has emerged as a particularly promising therapeutic strategy. The importance of exercise in PD management has been emphasized in several international clinical practice guidelines and expert consensus statements. The European Physiotherapy Guideline for Parkinson's Disease recommends exercise and physical therapy as key components of comprehensive PD care (Domingos et al., 2018). Similarly, recent physical therapy clinical practice guidelines and expert recommendations have highlighted exercise as a core therapeutic approach for maintaining mobility, improving balance, enhancing motor function, and promoting long-term health in individuals with PD (Osborne et al., 2022). Consistent with these recommendations, a growing body of evidence suggests that physical exercise can improve physical condition, motor performance, and functional capacity in people with PD (Radder et al., 2020). These findings underscore the importance of identifying the most effective exercise modalities for optimizing clinical outcomes in this population. Previous systematic reviews and meta-analyses have investigated the effects of various exercise modalities on motor and balance outcomes in individuals with PD, including aquatic exercise, aerobic exercise, and bicycling interventions (Gomes Neto et al., 2020; de Oliveira et al., 2021). Evidence from systematic reviews and meta-analyses suggests that bicycling-based interventions may improve motor symptoms, functional mobility, and quality of life in people with PD (Palmieri et al., 2024). Furthermore, recent scoping reviews have highlighted the growing interest in cycling as a rehabilitation strategy and summarized its potential benefits across a wide range of motor outcomes (Tiihonen et al., 2021). However, most existing reviews have focused on individual exercise modalities and have been limited to pairwise comparisons. Consequently, the relative effectiveness of different exercise interventions for improving balance and motor function in PD remains unclear. Network meta-analysis provides an opportunity to integrate direct and indirect evidence across multiple interventions and identify the most effective exercise strategies for this population.

## 2 Methods and analysis

### 2.1 Registration

This network meta-analysis was designed according to the guidelines for Preferred Reporting Items of Systems Review and Network Meta-Analysis (PRISMA-NMA) (Hutton et al., 2015), which are registered in the PROSPERO database (CRD42024584880).

### 2.2 Search strategy

Literature searches were independently and systematically conducted by two reviewers according to the PICO framework described in the Study Selection section. Electronic databases, including PubMed, Web of Science, Embase, Cochrane Library, and China National Knowledge Infrastructure (CNKI), were searched to identify relevant studies published up to August 1, 2025. A predefined search strategy was applied across all databases to ensure consistency and comprehensiveness. Any discrepancies encountered during the search and study identification process were resolved through discussion between the two reviewers or, when necessary, through consultation with a third reviewer. The search strategy uses Pubmed as an example, as shown in Supplementary Appendix 1.

### 2.3 Study selection

Study selection was conducted according to the PICOS framework (Participants, Interventions, Comparators, Outcomes, and Study Design) (Hutton et al., 2015). Two reviewers (Ying Li and Zhaojun Luo) independently screened the titles, abstracts, and full texts of all retrieved studies based on the predefined inclusion and exclusion criteria. Any disagreements regarding study eligibility were first resolved through discussion between the two reviewers; if consensus could not be reached, a third reviewer (Meiling Zhao) was consulted to make the final decision. In addition, Yanxia Tang supervised the entire selection process and verified the final list of included studies to ensure methodological rigor and consistency, see Table 1.

**Table 1:** Eligibility criteria for study selection.

Category	Inclusion criteria	Exclusion criteria
Population	Parkinson's disease was diagnosed in patients > 18 years of age, Hoehn and Yahr (H&Y) stages II–IV.	Patients with severe comorbidities such as severe hypertension, heart disease, or other serious systemic diseases (e.g., severe cardiovascular disease, malignant tumors, or other neurological disorders).
Interventions	Aerobic exercise (AE), Aquatic Exercise (AQE), Whole body vibration training (WBV), Virtual reality (VR), Treadmill training (TT), Resistance training (RT), Tai Chi (TC), Power Training (PT), Biofeedback Balance and Gait Training (BGT), Walking exercise (WE), Dance exercise (DE), Balance training (BT), Game training (GT), Baduanjin (BDJ), Pilates exercise (PE), Home exercise (HE), Yoga (YG), Cycling exercise (CE), Boxing exercise (BE), Robotic Assisted Gait Training (RAGT), Combined therapy (CT), Dual task training (DTT), Stretch exercise (SE), Five animal exercises (FAE), Other exercise (OE: other forms of exercise apart from those mentioned above), Fitness exercise (FE), Qigong (QG), Virtual reality balance training (VRB).	
Comparisons	Traditional Rehabilitation (TR), Control group (CON).	
Outcomes	The primary outcomes were the Berg Balance Scale (BBS) and the Timed Up and Go Test (TUG). The BBS is a widely used 14-item clinical scale for assessing balance performance and fall risk and has demonstrated good reliability and validity in individuals with PD (Qutubuddin et al., 2005). The TUG is a simple and reliable measure of functional mobility and dynamic balance and has shown good reliability and validity in people with PD (Mollinedo and Ma Cancela, 2020). Secondary outcomes included the Unified Parkinson's Disease Rating Scale Part III (UPDRS-III), a widely validated instrument for assessing the severity of motor symptoms in PD (Movement Disorder Society Task Force on Rating Scales for Parkinson's Disease, 2003).	
Study	Randomized controlled trial; published in English or Chinese.	

CON: usual care/waitlist/no-exercise. The UPDRS-III provides an assessment of global motor function. Each intervention is defined in Supplementary Appendix 2. Each result outcome measure is defined in Supplementary Appendix 3.

### 2.4 Data extraction

Data extraction was performed independently by two reviewers, capturing the first author, publication year, country, sample size, intervention modality, session duration, and total intervention period. Continuous outcomes were extracted and expressed as mean ± standard deviation (SD). In cases where an outcome was reported at multiple time points, data from the most recent follow-up assessment were prioritized. Additionally, to address potential overlap across intervention categories, each intervention was classified based on its primary training component or core modality, which best reflected the main therapeutic focus of the respective study.

### 2.5 Risk of bias assessment

The risk of bias was assessed independently by two reviewers and by a third reviewer using the tools provided by the Cochrane Collaboration (Higgins et al., 2011), including sequence generation, hidden assignment, blinding, incomplete outcome data, non-selective reporting of results, and other sources of bias. Each criterion was judged to have a low, unclear, or high risk of bias.

### 2.6 Assessment of transitivity across treatment comparisons

If the transitivity assumption can be demonstrated to hold, the use of network meta-analysis is justified. Transitivity requires that the distribution of potential effect modifiers—including study-level and patient-level covariates—is balanced across all pairwise comparisons (Cipriani et al., 2013; Salanti, 2012). To examine the transitivity assumption within our network, we selected two established potential effect modifiers—participant age and disease/treatment duration—as core indicators for assessing baseline characteristics. In addition to participant demographic baselines, design features of the clinical intervention protocols, such as treatment duration and session frequency, also serve as critical potential effect modifiers. To evaluate whether the distribution of these intervention characteristics was balanced across the different comparison groups, we performed a one-way analysis of variance (ANOVA) with “intervention” as the grouping variable, examining both the intervention duration and frequency reported in the included primary studies.

### 2.7 Data analysis

Network meta-analysis was conducted using the netmeta package in R version 4.2.1. Network diagrams illustrating the comparisons among different exercise interventions were generated using the “networkplot” function in STATA version 15.1. In the network diagrams, nodes represent different interventions, and edges represent direct comparisons between interventions. Consistency between direct and indirect evidence was assessed using the node-splitting approach (Rücker and Schwarzer, 2015). Pooled effect estimates and their 95% confidence intervals (95% CI) were calculated using a random-effects model. Mean differences (MDs) were used for outcomes measured on the same scale, whereas standardized mean differences (SMDs) were used for outcomes measured with different instruments. To evaluate the relative effectiveness of the exercise interventions and generate a ranking, higher values indicate a greater probability that an intervention is among the most effective treatment options. Sensitivity analyses were performed by sequentially excluding individual studies to assess the robustness and stability of the pooled results.

## 3 Results

### 3.1 Literature selection

**Figure 1:**
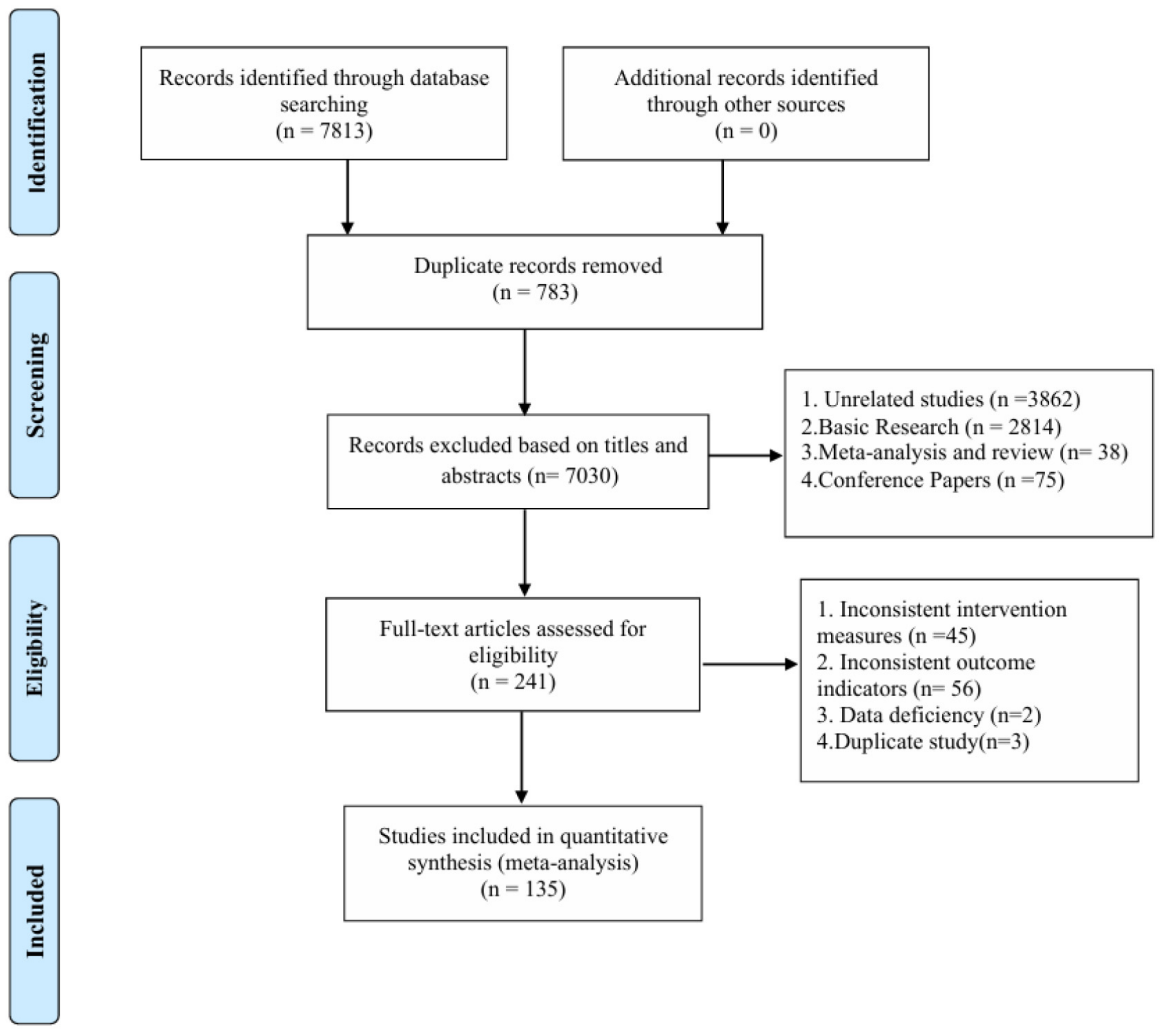
Flow chart.

After deleting duplicates, 7,813 records were retrieved, 783 duplicates were removed, 6789 articles with inconsistent titles were deleted, 106 articles with inconsistent titles were removed after reading the full text, and 135 articles were finally included (Supplementary Appendix 4:1–135). The research flow chart is shown in Figure 1.

### 3.2 Study and participant characteristics

**Figure 2:**
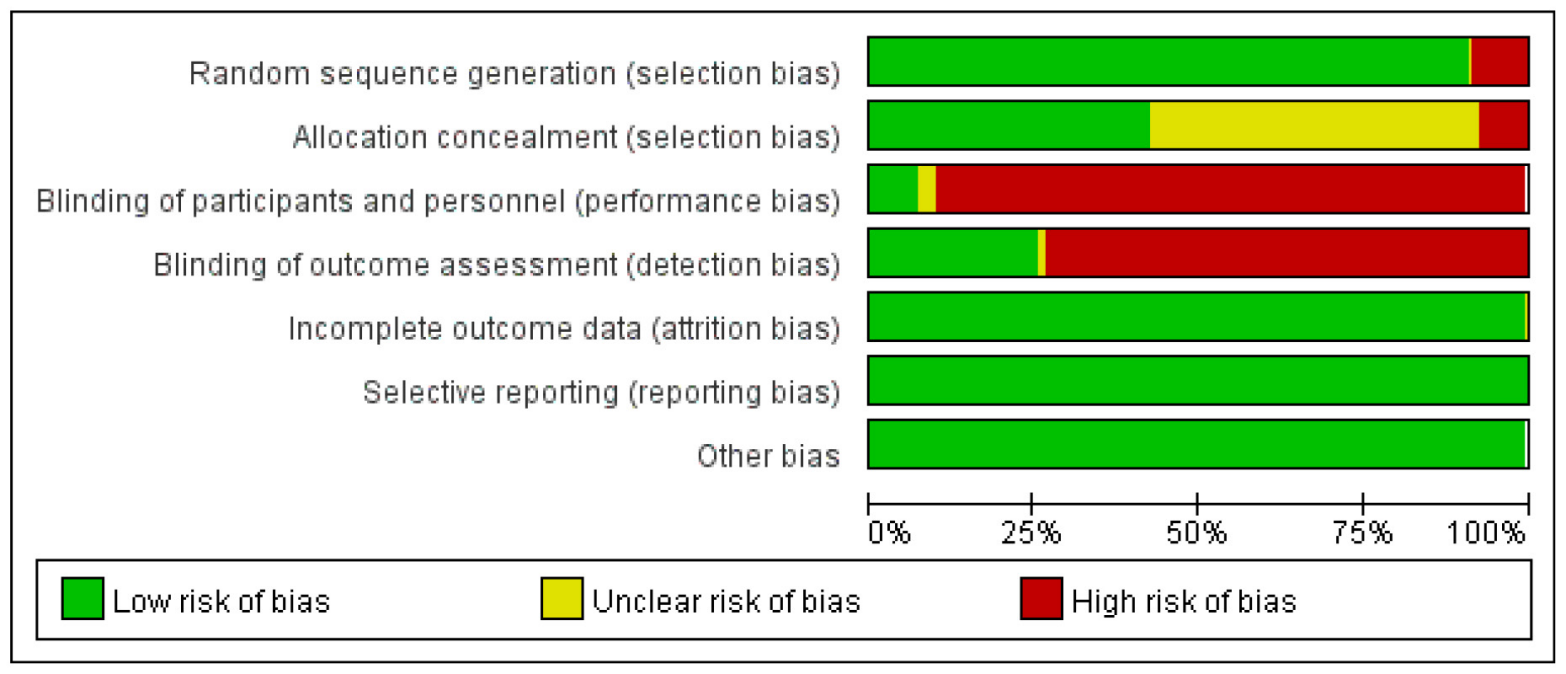
Distribution of risk of bias across included studies, as assessed by the Cochrane Risk of Bias tool.

The included studies, published between 2006 and 2024, compared the effects of 30 different forms of exercise on people with Parkinson's disease. The duration of intervention ranged from 8 days to 96 weeks. A total of 5,948 patients were reported. Of all the included studies, 110 reported the BBS, 72 reported the TUG test, and 63 reported the UPDRS-III. The mean age was 56.06–78.14 years, and the duration of illness was 27.0 months to 18.10 years. The duration of intervention ranged from 8 days to 96 weeks. A total of 5948 patients were reported. Of all the included studies, 110 reported BBS, 72 reported TUG, and 63 reported UPDRS-III. Mean age 56.06–78.14 years old, duration of illness 27.0M−18.10Y. The characteristics of the studies and participants are shown in Table 2 and Supplementary Appendix 4. An overall risk-of-bias judgement was assigned for each study. Studies with one or more key domains rated as high risk of bias were considered to have an overall high risk of bias. Studies without any high-risk ratings were classified as having an overall low risk or unclear risk of bias according to the highest level of concern identified across the assessed domains. The risk of bias assessment for each study is summarized in Supplementary Appendix 5 and Figure 2.

**Table 2:** Characteristics of the included studies.

Characteristics	BBS	TUG	UPDRS-III
Publication characteristics			
Total number of unique studies included	110	72	63
Publication year			
1991–2000	0	0	0
2001–2010	5	2	2
2011–2020	59	48	45
2021–2024	46	22	16
Study design characteristics			
Range of study sample size			
1–50	67	49	39
51–100	35	19	13
101–150	7	3	10
151–200	1	1	1
No. of intervention arms included			
2	107	69	61
3	3	3	2
4	0	0	0
No. of studies containing the following treatment nodes			
Aerobic exercise (AE)	1	0	2
Aquatic exercise (AQE)	6	5	5
Whole body vibration training (WBV)	2	1	4
Virtual reality (VR)	10	5	3
Treadmill training (TT)	3	2	1
Resistance training (RT)	7	4	2
Tai Chi (TC)	13	10	10
Power training (PT)	0	1	0
Biofeedback balance and gait training (BGT)	1	1	1
Control group (CON)	17	9	11
Walking exercise (WE)	5	4	2
Dance exercise (DE)	11	9	6
Balance training (BT)	17	5	4
Game training (GT)	5	1	0
Baduanjin (BDJ)	4	2	4
Pilates exercise (PE)	2	2	1
Home exercise (HE)	1	0	1
Yoga (YG)	2	2	1
Cycling exercise (CE)	1	0	1
Boxing exercise (BE)	1	1	0
Robotic assisted gait training (RAGT)	5	3	3
Combined therapy (CT)	18	9	14
Traditional rehabilitation (TR)	77	52	37
Dual task training (DTT)	2	2	1
Stretch exercise (SE)	0	1	1
Five animal exercises (FAE)	2	2	2
Other exercise (OE)	6	6	3
Fitness exercise (FE)	5	1	3
Qigong (QG)	0	4	4
Virtual reality balance training (VRB)	2	2	1
Time of intervention			
Unclear	5	4	2
8 days	0	1	1
20 days	1	0	0
3 weeks	1	0	1
4 weeks	21	10	9
5 weeks	2	2	2
6 weeks	10	4	8
7 weeks	3	0	0
8 weeks	22	16	11
9 weeks	1	0	0
10 weeks	7	8	3
11 weeks	0	1	0
12 weeks	25	20	13
16 weeks	2	1	2
20 weeks	0	0	0
24 weeks	7	5	6
48 weeks	2	0	3
96 weeks	0	0	1
During hospitalization	1	0	1
Intervention frequency			
Once a week	5	5	1
2 times/week	34	24	19
3 times/week	17	10	10
2–3 times/week	0	1	1
4 times/week	5	3	3
5 times/week	23	15	15
6 times/week	2	1	0
Unclear	16	9	11
3 days a week	1	1	1
3–5 times a week	1	0	0
5 days a week	4	2	1
5 times in 5 days	1	1	1
Every day	1	0	0
Patient characteristics			
Range of mean age (years)	56.06–78.14	56.08–74.05	56.06–75.0
Duration of diagnosis (year/month)	27.0 M−18.10Y	1.72Y− 18.10Y	36 M−10.3Y

### 3.3 Transitivity

Regarding baseline age, 115 studies (accounting for 80.42% of the total included data) showed no statistically significant difference from the overall mean distribution (*t-*test, *p* > 0.05). For disease/treatment duration, 109 studies (representing 76.22% of the total included data) exhibited no statistically significant difference from the overall mean distribution (*t-*test, *p* > 0.05). Concerning intervention duration, no statistically significant differences were observed among the various intervention comparison groups (F = 0.651, *p* = 0.896). However, for intervention frequency, analysis of variance revealed statistically significant differences across comparison groups (F = 1.722, *p* = 0.031). Overall, the transitivity assumption for this network meta-analysis was considered valid, thereby satisfying the prerequisite conditions for conducting network meta-analysis, (Supplementary Appendix 10).

### 3.4 BBS

**Figure 3:**
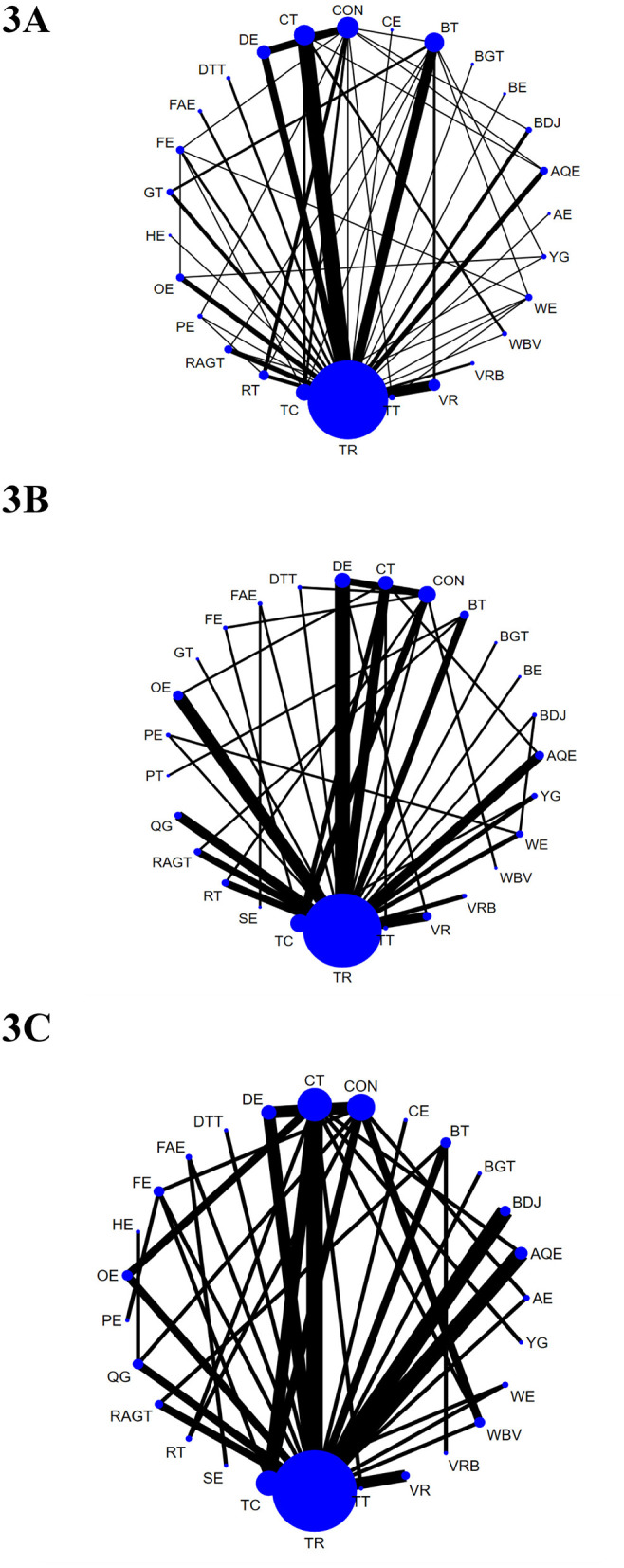
Network diagram of BBS, TUG, and UPDRS-III in patients with Parkinson's disease. The node size represents the number of times the exercise appears in any comparison about that treatment, and the width of the edge represents the total sample size in the comparison it connects. Aerobic exercise (AE), Aquatic Exercise (AQE), Whole body vibration training (WBV), Virtual reality (VR), Treadmill training (TT), Resistance training (RT), Tai Chi (TC), Power Training (PT), Biofeedback Balance and Gait Training (BGT), Control group (CON), Walking exercise (WE), Dance exercise (DE), Balance training (BT), Game training (GT), Baduanjin (BDJ), Pilates exercise (PE), Home exercise (HE), Yoga (YG), Cycling exercise (CE), Boxing exercise (BE), Robotic Assisted Gait Training (RAGT), Combined therapy (CT), Traditional Rehabilitation (TR), Dual task training (DTT), Stretch exercise (SE), Five animal exercises (FAE), Other exercise (OE), Fitness exercise (FE), Qigong (QG), Virtual reality balance training (VRB).

A total of 110 studies evaluated BBS, involving 5,694 participants. We included the following 27 exercise measures in our network meta-analysis (Figure 3A and Supplementary Appendix 11). Our results show that Dance exercise (DE) improves BBS in patients with Parkinson's disease better than Control group (CON) (MD, 6.25; 95% CI, 2.81 to 9.69) and Traditional Rehabilitation (TR) (MD, 7.56; 95% CI, 3.89 to 11.23). SUCRA ranking results for BBS showed that DE ranked highest (88.1%), followed by WE (82.3%) and RAGT (71.8%) (Supplementary Appendix 6.1). In addition, we performed the Egger's test to assess publication bias (*p* = 0.015) (Supplementary Appendix 7.1). For BBS, the global consistency test showed no significant inconsistency within the network (Q = 17.85, df = 19, *P* = 0.5327). The estimated between-study heterogeneity was moderate (τ^2^ = 13.78), indicating an acceptable level of variability across the included studies (Supplementary Appendix 8). We conducted a direct comparison of exercise interventions (Supplementary Appendix 9.1).

### 3.5 TUG

A total of 72 studies evaluated TUG, involving 3,437 participants. We included the following 27 exercise measures in the network meta-analysis (Figure 3B and Supplementary Appendix 12). Our results show that Biofeedback Balance and Gait Training (BGT) is better than Control group (CON) (MD, −10.47; 95% CI, −20.13 to −0.81), Traditional Rehabilitation (TR)(MD, −10.60; 95% CI, −20.05 to −1.15), Stretch exercise (SE) (MD, −14.30; 95% CI, −26.75 to −1.85), Virtual reality balance training (VRB) (MD, −12.85; 95% CI, −23.40 to −2.29), and yoga (YG) (MD, −13.29; 95% CI, −23.97 to −2.61) in improving TUG scores. SUCRA ranking results for TUG showed that BGT ranked highest (95.1%), followed by PE (89.8%) and VR (79.0%) (Supplementary Appendix 6.2). In addition, we performed the Egger test to assess publication bias (*p* = 0.537) (Supplementary Appendix 7.2). For TUGT, no significant inconsistency was detected in the network (Q = 20.24, df = 16, *P* = 0.2096). The estimated heterogeneity was relatively low (τ^2^ = 4.85), suggesting good consistency and limited between-study variability (Supplementary Appendix 8). We made a direct comparison of exercise interventions (Supplementary Appendix 9.2).

### 3.6 UPDRS-III

A total of 63 studies evaluated UPDRS-III, involving 3,452 participants. We included the following 27 exercise measures in the network meta-analysis (Figure 3C and Supplementary Appendix 13). The results showed that PE was superior to CON (MD, −13.47; 95% CI, −25.98 to −0.96) in improving UPDRS-III scores. QG was superior to TR (MD, −6.24; 95% CI, −10.87 to −1.60), RT (MD, −8.21; 95% CI −16.10 to −0.33) and CON (MD, −8.89; 95% CI −14.35 to −3.42) in improving UPDRS-III scores. SUCRA ranking results for UPDRS-III showed that PE ranked highest (86.4%), followed by QG (77.5%) and AE (74.5%) (Supplementary Appendix 6.3). In addition, we assessed publication bias using the Egger test (*p* = 0.656) (Supplementary Appendix 7.3). For UPDRS-III, the global consistency test indicated no evidence of significant inconsistency (Q = 17.81, df = 16, *P* = 0.3353). The estimated heterogeneity was moderate (τ^2^ = 8.49) (Supplementary Appendix 8). We made a direct comparison of exercise interventions (Supplementary Appendix 9.3).

### 3.7 Sensitivity analysis

To assess the robustness of the pooled results, we conducted a sensitivity analysis using Review Manager 5.3 software. The findings of the meta-analysis remained consistent after sequentially excluding each individual study through the leave-one-out method, indicating that our conclusions are robust.

## 4 Discussion

First, this study represents the most comprehensive and systematic comparative network meta-analysis to date on the effects of exercise on balance in patients with Parkinson's disease. We included 135 studies comprising 5,948 patients and categorized interventions into 30 distinct types of exercise. By conducting both direct and indirect comparisons, we aimed to identify the most effective exercise modalities for improving balance and motor function in Parkinson's disease. Our findings indicated that Dance Exercise (DE) was more effective than the Control group (CON) and Traditional Rehabilitation (TR) in improving BBS scores; Biofeedback Balance and Gait Training (BGT) showed superior performance in reducing TUG testing time compared with Stretch Exercise (SE), CON, TR, Virtual Reality Balance Training (VRB), and Yoga (YG); and Pilates Exercise (PE) was more effective than CON in improving UPDRS-III scores. Overall, these results provide novel and comprehensive evidence-based support for exercise interventions in Parkinson's disease, with clear clinical significance.

The BBS assesses static and functional balance and is most suitable for individuals with mild to moderate Parkinson's disease. It may show ceiling effects in patients with relatively preserved balance, so the observed superiority of certain exercise interventions is particularly relevant to this group (King et al., 2012). Dance exercise (DE) demonstrates superior effectiveness in improving BBS scores compared to control group (CON) and traditional rehabilitation (TR). In addition, we referred to the established benchmark, the minimum clinically important difference (MCID) of balance in People with PD (Godi et al., 2020). In our research, several intervention measures, such as dancing, produced effects that exceeded this threshold compared with the control group, indicating that their benefits were not only statistically significant but also meaningful in clinical practice. The BBS comprehensively evaluates functional balance, providing insights into a patient's ability to strategically reposition their center of gravity (Steffen et al., 2002; Pickenbrock et al., 2016). As a form of exercise therapy, dance is simple to implement, highly operable (Canning et al., 2014), and entertaining, making it well-suited for the rehabilitation of people with PD (Rizos et al., 2014). The diverse movements in dance—including forward, backward, and sideways steps, rotations, jumps, and single-leg stances—require continuous maintenance of body balance (Earhart, 2009; Frisaldi et al., 2021). Evidence suggests that dance can strengthen neural connections associated with motor control and memory (de Natale et al., 2017). This neural facilitation contributes to the alleviation of core motor symptoms in Parkinson's disease, such as bradykinesia, rigidity, and tremor, while simultaneously enhancing patients' flexibility, muscle tone, and postural balance (Duncan and Earhart, 2012; Hackney et al., 2007). Furthermore, partner dancing demands timely responses to a partner's movements, emphasizing dynamic stability training (Federici et al., 2005). This may lead to improved joint flexibility, alleviation of movement disorders, and enhanced balance function (Hao and Yuxiu, 2016). However, it is worth noting that the BBS primarily evaluates balance, postural control, and weight-shifting ability, which are directly targeted by dance-based training. In contrast, the TUG assesses functional mobility and gait performance, which depend more on lower-limb strength and gait efficiency. Consequently, dance interventions may yield greater improvements in balance than in TUG performance. This contextualization helps to transform our statistical results into practical suggestions for exercise prescriptions. Nevertheless, given the remaining limitations of the current evidence, we recommend that future large-scale, preregistered randomized controlled trials be conducted to further validate the efficacy of dance therapy on balance ability in patients with PD. However, it is worth noting that dance interventions may carry a higher fall risk. Therefore, patients should perform dance exercises under professional supervision, select movements and intensity according to their balance ability, and ideally practice in a safe environment or with supportive equipment to minimize the risk of falls.

The TUG evaluates functional mobility, dynamic balance, and fall risk and can be applied across a broad spectrum of Parkinson's disease severity. However, it is particularly useful for identifying mobility limitations and fall risk in individuals with mild-to-moderate disease, where changes in gait and transitional movements can be captured sensitively (Winser et al., 2019). Biofeedback Balance and Gait Training (BGT) shows greater efficacy than Stretch Exercise (SE), Control Group (CON), Traditional Rehabilitation (TR) in improving TUG performance. The TUG primarily assesses motor function and the severity of freezing of gait (Casamassima et al., 2014). Biofeedback delivers real-time visual (van den Heuvel et al., 2014), auditory (Yen et al., 2011; Ginis et al., 2016), or vibrotactile feedback (Lee et al., 2015) to participants, enabling them to recognize and correct movement errors, thereby improving attention and engagement during training. This process allows participants to correct their movements, thereby increasing attention and motivation. Concurrently, gait and balance exercises improve lower limb strength and stability. By applying external forces to perturb balance, patients learn to adjust their posture to restore stability, thereby enhancing neuromuscular coordination and dynamic balance, which is directly reflected in improved TUG scores. Preliminary evidence suggests that BGT may have the potential to improve balance in patients with Parkinson's disease. However, it is crucial to note that this finding is primarily derived from a single trial, and thus its definitive efficacy needs to be verified in future large-scale studies.

UPDRS-III is a comprehensive measure of motor symptom severity and is applicable across all stages of Parkinson's disease (Skorvanek et al., 2017). Our study found that Pilates exercise (PE) and Qigong (QG) are more effective than CON in improving UPDRS-III in Parkinson's patients. UPDRS-III serves as an indicator of global motor function in individuals with PD (Ramsay et al., 2020). The mechanism of Pilates exercise (PE) involves the coordinated activation of both agonist and antagonist muscle groups to promote postural stability, thereby improving balance, motor coordination, muscle strength, endurance, and flexibility (Kloubec, 2010). These combined benefits ultimately lead to enhanced global motor function in People with PD. QG is a specialized movement therapy originating from Traditional Chinese Medicine (Putiri et al., 2017; Guo et al., 2019). As an integrated practice, it combines specific postures, breathing techniques, and meditation to regulate “Qi” (vital energy) and harmonize the Yin-Yang balance within the body, thereby clearing potential blockages in energy flow and promoting overall health (Guo et al., 2019; La Forge, 2016; Matos et al., 2015). From a kinematic perspective, QG practice requires coordinated shifting of the body's center of gravity guided by upper limb movements (Li et al., 2022). It emphasizes slow motion, active trunk control in space, and multi-directional weight transfer. Furthermore, most QG movements constitute closed-chain exercises for the lower limbs, a characteristic that aids in correcting inadequate heel strike and insufficient knee extension during the gait cycle (Liu et al., 2016). For patients with PD, QG enhances plantar support and postural stability, effectively improving gait parameters such as increased stride length and reduced walking impairment (Li et al., 2022). Evidence indicates that the stretching components in QG can reduce the percentage of double-limb support time during single-task performance (Li et al., 2022). Multiple studies have shown that static stretching combined with lower muscle tension improves balance, alleviates motor symptoms, enhances mobility, and increases backward walking speed in Parkinson's patients (Rawson et al., 2019; Mak et al., 2017). Collectively, these effects contribute to improved overall motor function in this population.

## 5 Strengths and limitations

However, several limitations should be acknowledged, and the results should be interpreted with caution: First, regarding the methodological assessment, given the large number of included studies, re-evaluating the risk of bias using the currently recommended RoB-2 tool was not feasible; therefore, this study utilized the original Cochrane Risk of Bias tool (RoB 1). Although the risk of bias of individual studies was assessed, the overall certainty of evidence was not evaluated using the GRADE approach. Future studies should incorporate GRADE assessments to provide a more comprehensive evaluation of the strength and reliability of the evidence. Furthermore, the overall quality of blinding implementation in the included literature was suboptimal, which may introduce a certain degree of bias. Second, concerning the reporting of intervention details, we did not account for variations in intervention frequency (Borde et al., 2015; Milanović et al., 2015). Additionally, although previous studies indicate that exercise intensity is a crucial factor influencing intervention efficacy, standards for intensity vary across different exercise types, and many included studies failed to report it. Such discrepancies in interventions may affect the generalizability of our results, and caution is warranted when interpreting the observed effect sizes. Third, regarding sample and study characteristics, several top-ranked interventions were primarily based on small-scale trials. The limited sample size may lead to instability in effect estimates, thereby affecting the robustness and clinical applicability of the conclusions. Moreover, there was heterogeneity in the baseline characteristics of patients across the included original studies, particularly concerning the Hoehn and Yahr stage, which may limit the extrapolation of our findings. Fourth, regarding outcome measure evaluation, apart from the BBS, there is a lack of established MCID values for other commonly used balance and motor function assessment tools in the PD population. This limitation highlights a critical direction for future research: establishing MCID thresholds for these outcome measures to more accurately evaluate the actual clinical value of interventions for patients. In conclusion, future large-scale, multicenter randomized controlled trials conducted in more homogeneous populations are still needed to validate our findings. Finally, although heterogeneity was assessed using the I^2^ statistic, additional measures, such as tau-squared (τ^2^), which reflects between-study variance, and prediction intervals, which provide information on the potential range of treatment effects in future studies or real-world clinical settings, were not evaluated. Therefore, the assessment of heterogeneity and the real-world variability of the study findings may not have been fully comprehensive. Future network meta-analyses should incorporate multiple heterogeneity measures to provide a more thorough evaluation of between-study variability and enhance the interpretability and clinical applicability of the findings.

## 6 Conclusion

Compared with the CON and TR, DE, and BGT demonstrated superior efficacy in improving balance function (BBS, TUG) in patients with PD. Additionally, PE and QG showed greater benefits than CON in enhancing overall motor function, as assessed by the UPDRS-III. These findings highlight that distinct exercise interventions provide specific and clinically meaningful improvements across different functional domains. Consequently, a combination or personalized exercise regimen may represent the optimal approach for individuals with PD. For instance, integrating modalities such as dance, biofeedback balance training, and Qigong throughout the week could yield comprehensive and synergistic benefits. Crucially, clinical exercise prescriptions should prioritize patient preference and adherence, tailoring the regimen to the individual's interests and capabilities. Future large-scale, high-quality randomized controlled trials are warranted to validate these combined effects and further refine personalized exercise prescriptions for PD management.
