# Supplementary material for: Which exercise intervention is most promising for Parkinson's balance? A network meta-analysis
Source: Front Aging Neurosci. 2026 Jul 15;18:1879017. doi: 10.3389/fnagi.2026.1879017 (PMC13416450; doi:10.3389/fnagi.2026.1879017)
Supplement: Supplementary file 1 [file Data_Sheet_1.zip › Supplementary materials/Appendix 4-Included literature.docx]

| **Author** | **Publish**  **Year** | **Country** | **measure** | **Age**  **(Mean ± SD)** | **Number**  **(male/** **female)** | **duration of diagnosis**  **(year/month)** | **Intervention**  **time** | **Intervention**  **frequency** | **Outcomes** |
| --- | --- | --- | --- | --- | --- | --- | --- | --- | --- |
| Cheng^[1]^ | 2019 | China | VR/TR | 59.2±7.3/58.6±7.5 | 17/23 | 6.1±1.4 /  6.2±1.7 | 8weeks | 2 times a week | ①② |
| Liu^[2]^ | 2020 | China | BT/TR | 60.9±7.20/63.90±5.82 | 23/29 | 7.76±3.99/  8.10±3.42 | 4 weeks | 5 days a week | ① |
| Ding^[3]^ | 2022 | China | RAGT/TR | 74.05±7.04/71.35±8.56 | 25/15 | 5.95±4.42/  6.30±4.27 | 8 weeks | 5 days a week | ①②③ |
| Cao^[4]^ | 2021 | China | FAE/TR | 62.45±5.13/66.48±5.15 | 41/21 | 6.10±0.87/  5.71±1.10 | 8 weeks | Once a week | ①② |
| Guan^[5]^ | 2016 | China | TC/TR | 66.48±5.15/ 66.45±5.13 | 33/29 | 4.43±3.17/  4.28±3.25 | 12 weeks | 4 times a week | ① |
| Shen^[6]^ | 2022 | China | CT/TR | 66.48±5.15 | 35/27 | NA | 24 weeks | 2 times a week | ①② |
| Zhang^[7]^ | 2021 | China | BT/TR | 62.33±8.58/65.1±11.45 | 38/42 | 6.28±3.62/  7.67±6.67 | 6 weeks | 5 times a week | ①②③ |
| Tang^[8]^ | 2018 | China | RT/TR | 62.33±8.58/65.1±11.45 | 43/19 | NA | 12 weeks | 2 times a week | ① |
| Volpe^[9]^ | 2016 | Italy | AQE/TR | 70.6±7.8/70±7.8 | 19/11 | 9.4±7.5/  9±7.0 | 8 weeks | NA | ①②③ |
| Picelli^[10]^ | 2012 | Italy | RAGT/TR | 68.3 | 20/14 | 7.5±3.45 | 4 weeks | 3 days a week | ①②③ |
| Natale^[11]^ | 2017 | Italy | DT/TR | 66.0±9.15/70.0±3.16 | 11/16 | 6.0 ±2.07/  6.33± 2.25 | 10 weeks | 2 times a week | ①②③ |
| Guadarrama^[12]^ | 2020 | Mexico | WBV/CT/TR | 63.5±9.9 | 27/18 | NA | 20 days | 3 times a week | ① |
| Gao^[13]^ | 2014 | China | TC/TR | 69.54±7.32/68.28±8.53 | 50/26 | 9.15±8.58/  8.37±8.24 | 12 weeks | 3 times a week | ①②③ |
| Zhang^[14]^ | 2015 | China | TC/CT | 66.00±11.80/64.35±10.53 | 24/16 | 6.80±5.43/  4.85±3.72 | 12 weeks | 2 times a week | ①②③ |
| Hashimoto^[15]^ | 2015 | Japan | DE/TR/CON | 67.9±7.0/62.7±14.9 | 12/34 | 6.3±4.6/  7.8±6.2/  6.9±4.0 | 12 weeks | Once a week | ①② |
| Lee^[16]^ | 2015 | Korea | GT/TR | 68.4±2.9/70.1±3.3 | 10/10 | NA | 6 weeks | 5 times a week | ① |
| Xiao^[17]^ | 2016 | China | BDJ/TR | 67.53±8.56 | 67/29 | 5.45±3.61/  6.15±2.63 | 24 weeks | 4 times a week | ①②③ |
| Feng^[18]^ | 2019 | China | VR/TR | 67.47±4.79/66.93±4.64 | 17/13 | 7.07±1.44/  6.60±1.45 | 12 weeks | 5 times a week | ①②③ |
| Cakit^[19]^ | 2007 | Turkey | TT/CON | 71.8±6.4 | 16/15 | 5.58±2.9 | 8 weeks | NA | ① |
| Picelli^[20]^ | 2015 | Italy | RAGT/BT | 68.2±9.2/ 69.7±7.2 | 48/18 | 7.5±5.6/  8.3±4.1 | 4 weeks | 3 times a week | ①②③ |
| Clerici^[21]^ | 2019 | Italy | AQE/CT | 67±9 | 39/13 | NA | 4 weeks | 5 times in five days | ①②③ |
| Palamara^[22]^ | 2017 | Italy | AQE/TR | 70.9±5.7/70.8±5.3 | 20/14 | NA | 4 weeks | NA | ①②③ |
| Smania^[23]^ | 2010 | Italy | BT/TR | 67.64±7.41/ 67.26±7.18 | 29/26 | 10.39±4.76/  8.63/5.39 | 7 weeks | 3 times a week | ① |
| Xiao^[24]^ | 2016 | China | BDJ/TR | 67.8±9.4 | NA | NA | 24 weeks | 4 times a week | ①②③ |
| Carvalho^[25]^ | 2015 | Brazil | PT/TR/AE | 64.8±11.9/64.1±9.9/  62.1±11.7 | 16/6 | 6.6±1.5/  6.0±2.6/  4.3±2.8 | 12 weeks | 2 times a week | ①②③ |
| Vieira^[26]^ | 2020 | Brazil | RT/CON | 64.7±1.8/64.4±3.7 | 30/10 | 5.7±0.8/  7.2±1.9 | 9 weeks | 2 times a week | ① |
| Michels^[27]^ | 2018 | America | DT/CONP | 66.44/75.50 | NA | 3.76±2.88/  5.94±3.61 | 12 weeks | NA | ①②③ |
| Çoban^[28]^ | 2021 | Turkey | PE/TR | 58.85±8.09/ 60.75±7.62 | 19/21 | 5.32±6.23/  5.35±3.33 | 8 weeks | 2 times a week | ①② |
| Khuzema^[29]^ | 2020 | Brazil | TC/YG/BT | 72±5.22/68.11±4.23/  70.89±6.01 | 19/8 | NA | 8 weeks | NA | ①② |
| Pompeu^[30]^ | 2012 | India | GT/BT | NA | NA | NA | 7 weeks | 2 times a week | ① |
| Ribas^[31]^ | 2017 | Brazil | GT/TR | 61.70±6.83/60.20±11.29 | 8/12 | 6.5±4/  7±2.79 | 12 weeks | 2 times a week | ① |
| Shih^[32]^ | 2016 | China | GT/TR | 67.5±9.96/ 68.8±9.67 | 16/4 | 4.03±3.74/  5.22±4.85 | 8 weeks | 2 times a week | ①② |
| Cugusi^[33]^ | 2015 | Italy | WE/TR | 68.1±8.7/ 66.6±7.3 | 16/4 | 7±2/  7±4 | 12 weeks | 2 times a week | ①②③ |
| Kurt^[34]^ | 2017 | Turkey | TC/TR | 62.41±6.76/63.61±7.18 | 24/16 | NA | 5 weeks | 5 times a week | ①②③ |
| Bang^[35]^ | 2016 | Korea | WE/TT | 58.30±7.71/ 60.60±6.74 | 9/11 | 18.10±6.77/  17.98±3.28 | 4 weeks | 5 times a week | ①② |
| Hackney^[36]^ | 2009 | America | DT/CON | 68.8±6.39/68.8±9.68 | 34/14 | NA | 10 weeks | 2 times a week | ①② |
| Solla^[37]^ | 2018 | Italy | DT/TR | 67.8±5.9/ 67.1±6.3 | 13/20 | 4.4 4.5/  5±2.9 | 12 weeks | 2 times a week | ①② |
| Lee^[38]^ | 2018 | Korea | CT/CON | 65.8±7.2/65.7±6.4 | 17/24 | 4.5±3.3/  4.4±3.0 | 8 weeks | 2 times a week | ① |
| Cherup^[39]^ | 2021 | America | YG/OE | 69.8±7.3/ 71.4±12.1 | 21/12 | 1.7±0.5/  2±0.8 | 12 weeks | 2 times a week | ① |
| TILLMANN^[40]^ | 2020 | Brazil | DT/CON | 67.6±10.9 /65.30±10.5 | 16/4 | NA | 12 weeks | 2 times a week | ① |
| Kashif^[41]^ | 2022 | Pakistan | VR/TR | 63.86±4.57/62.32±4.61 | 22/22 | 6.23±1.85/  6.55±1.68 | 12 weeks | 3 times a week | ① |
| Pazzaglia^[42]^ | 2019 | Italy | VR/TR | 72/70 | 25/26 | NA | 6 weeks | 3 times a week | ① |
| Silva^[43]^ | 2018 | Brazil | AQE/CON | 63.12±13.61/64.23±13.45 | 14/11 | NA | 10 weeks | 2 times a week | ①② |
| Kashif^[44]^ | 2024 | Pakistan | VR/TR | 63.20±4.85/61.95±4.62 | 33/27 | 6.65±1.59/  6.25±1.77 | 16 weeks | NA | ①③ |
| Volpe^[45]^ | 2013 | Italy | DT/TR | 61.6±4.5/65.0±5.3 | 13/11 | 9.0±3.6/  8.9±2.5 | 24 weeks | Once a week | ① |
| Chen^[46]^ | 2021 | Brazil | RT/CON | 63.4±6.9/63.6±7 | 53/21 | 7.6±6/  8.4±5.9/  9.6±4.8 | 12 weeks | 2 times a week | ①②③ |
| Ventura^[47]^ | 2016 | America | DT/CON | 71.8±3.6/70.4±5.5 | 2/13 | 6.1±3.1/  4.3±2.6 | 10 weeks | Once a week | ①② |
| Ashburn^[48]^ | 2006 | Britain | HE/TR | 72.7±9.6/71.6±8.8 | 86/56 | 7.7±5.8/  9.0±5.8 | 8 weeks | Every day | ① |
| Yang^[49]^ | 2016 | China | VR/BT | 72.5±8.4/75.4±6.3 | 9/14 | 9.4±3.6/  8.3±4.1 | 6 weeks | 2 times a week | ①②③ |
| Picelli^[50]^ | 2013 | Italy | RAGT/TT/TR | 68.50±10.10/68.80±7.72/  67.55±7.08 | 23/37 | 6.52±5.30/  6.99±6.17/  6.79±6.30 | 4 weeks | 3 times a week | ① |
| Gandolfi^[51]^ | 2017 | Italy | VR/BT | 67.45±7.18/69.84±9.41 | 51/25 | 6.16±3.81/  7.47±3.90 | 7 weeks | 3 times a week | ① |
| Carpinella^[52]^ | 2017 | Italy | BGT/TR | 73.0±7.1/75.6±8.2 | 23/14 | 7.5±3.2/  10.3±5.7 | NA | 3 times a week | ①②③ |
| Zhang^[53]^ | 2024 | China | OE/TR | 68.05±6.24/68.47±7.40 | 20/19 | 4.80±1.89/  5.21±5.00 | 4 weeks | 3 times a week | ①② |
| Wu^[54]^ | 2024 | China | DTT/TR | 64.37±8.06/63.51±7.28 | 67/42 | 3.69±1.02/  3.45±0.86 | NA | 5 times a week | ①②③ |
| Volpe^[55]^ | 2014 | Italy | AQE/TR | 68±7/66±8 | NA | 7.5±5.1/  7.6±4.63 | 8 weeks | 5 times a week | ①② |
| Qutubuddin^[56]^ | 2012 | America | CE/TR | NA | 13/10 | NA | 8 weeks | 2 times a week | ①③ |
| Lin^[57]^ | 2016 | China | VRB/TR | 61.4±8.2/62.1±6.3 | 22/11 | 6.1±2.3/  6.4±1.9 | 4 weeks | 5 times a week | ①② |
| Sun^[58]^ | 2020 | China | CT/TR | 61.43±7.34/62.54±6.98 | 40/20 | 6.17±2.39/  6.44±1.97 | 4 weeks | 5 times a week | ① |
| Chen^[59]^ | 2017 | China | VR/TR | 64.65±5.06/62.09±6.11 | 26/20 | 7.76±2.32  8.04±2.10 | 6 weeks | 5 times a week | ①②③ |
| Wang^[60]^ | 2022 | China | DT/CON | 65.61±6.76/62.61±5.08 | 9/27 | 5.06±1.26/  5.11±1.32 | 12 weeks | 2 times a week | ①② |
| Gao^[61]^ | 2022 | China | TC/FE/CON | 64±5/65±8/ 63±6 | 24/39 | NA | 16 weeks | 3 times a week | ①②③ |
| Lei^[62]^ | 2021 | China | CT/TR | 56.8±3.4/58.3±5.5 | 40/40 | 5.26±1.58/  5.53±2.11 | NA | 6 times a week | ①② |
| Zeng^[63]^ | 2020 | China | WBE/CT | 63.27±4.54/ 62.36±3.10 | 18/15 | 6.81±1.60/  6.18±1.32 | 6 weeks | 5 times a week | ①③ |
| Zhu^[64]^ | 2022 | China | RT/TR | 67.8±4.6/69.3±5.1 | 46/32 | 3.7±1.6/  3.3±1.4 | 6 weeks | 5 times a week | ① |
| Wu^[65]^ | 2023 | China | RT/TR | 66.89±1.26/66.37±1.01 | 20/20 | NA | 4 weeks | 5 days a week | ①② |
| Cui^[66]^ | 2022 | China | OE/TR | 63.67±5.45/65.58±5.38 | 13/11 | 4.83±2.69/  5.75±2.00 | 12 weeks | 2 times a week | ①②③ |
| Li^[67]^ | 2021 | China | VR/TR | 57.33±8.73/ 57.04±7.15 | 28/20 | NA | 8 weeks | 3 times a week | ①② |
| Liu^[68]^ | 2023 | China | BDJ/TR | 63.69±5.04/64.05±4.54 | 82/34 | NA | During hospitalization | NA | ①③ |
| Zhang^[69]^ | 2022 | China | BT/TR | 67.00±6.17/ 71.89±8. 06 | 19/19 | NA | 4 weeks | 5 times a week | ① |
| Li^[70]^ | 2017 | China | TC/TR | NA | NA | NA | 12 weeks | 4 times a week | ① |
| Lu^[71]^ | 2017 | China | TC/TR | 67. 75±6. 84/ 68.20±7.32 | 10/6 | 2. 41±0. 48/  2.02±0.52 | 8 weeks | 5 times a week | ①③ |
| Zhang^[72]^ | 2022 | China | FAE/TR | 67.37±6.53 | 68/52 | 8.12±6.15 | 6 weeks | NA | ①③ |
| Han^[73]^ | 2021 | China | CT/TR | 68.60±7.37/67.88±8.63 | 49/51 | 36(24, 120)/  36(24, 111) M | 8 weeks | 3 times a week | ①③ |
| Ding^[74]^ | 2023 | China | TC/TR | 69.34±4.02/69.12±4.06 | 50/34 | 4.12±0.38/  4.25±0.42 | 4 weeks | NA | ① |
| You^[75]^ | 2020 | China | TC/CT | 68.81±5.02/ 68.49±5.27 | 37/33 | 4.21±0.24/  4.17±0.35 | 24 weeks | 2 times a week | ①③ |
| Sun^[76]^ | 2022 | China | DTT/TR | 62.9± 4.7/63.1±4.6 | 49/37 | 5.35±0.84/  5.41±0.89 | 24 weeks | 2 times a week | ① |
| Yang^[77]^ | 2018 | China | BT/TR | 66.48±6.71/67.21±7.74 | 139/107 | 8.58±3.21/  9.11±3.75 | 4 weeks | 5 times a week | ①②③ |
| Zhao^[78]^ | 2020 | China | FE/TR | 61.08±4.41/60.48±5.46 | 30/20 | NA | 4 weeks | 6 times a week | ① |
| Tang^[79]^ | 2020 | China | FE/WE | 66.85±9.27/67.13±8.94 | 21/21 | NA | 4 weeks | 3-5 times a week | ① |
| Zhang^[80]^ | 2019 | China | CT/CON | 65.06±4.905/ 63.94±5.068 | 13/21 | 2.59±1.58/  2.47±1.12 | 48 weeks | 2 times a week | ①③ |
| Peng^[81]^ | 2023 | China | WE/BT | 78.14±10.06/74.00±9.26 | 23/20 | 8.25±3.69/  8.02±3.62 | 12 weeks | 3 times a week | ① |
| Zhang^[82]^ | 2023 | China | CT/TR | 61.13±5.12/61.83±5.53 | 30/30 | 2.22±0.53/  2.05±0.52 | 8 weeks | 5 times a week | ①②③ |
| Xi^[83]^ | 2022 | China | OE/TR | 67.41±4.42/67.02±4.52 | 33/33 | 4.00±2.06/  3.36±1.52 | 8 weeks | 5 times a week | ①② |
| Wang^[84]^ | 2023 | China | TC/CON | 72.07±8.33/67.13±8.33 | 18/12 | 4.27±3.31/  5.87±2.72 | 24 weeks | 3 times a week | ①②③ |
| Wang^[85]^ | 2022 | China | FE/OE | 63.24±4.53/ 63.14±4.58 | 50/60 | 5.25±0.74/  5.35±0.82 | NA | NA | ① |
| Shi^[86]^ | 2021 | China | CT/TR | 67.89±4.63/67.48±4.52 | 68.61 | 6.58±1.71/  6.47±1.65 | 8 weeks | 4 times a week | ①③ |
| Lu^[87]^ | 2023 | China | CT/TR | 67.25±9.06/ 66.50±6. 24 | 27/13 | 4. 25±1. 81/  3. 65±1. 85 | 3 weeks | 5 times a week | ① |
| Zhou^[88]^ | 2023 | China | CT/TR | 68.52±4.39 68.71±4.45 | 64/36 | 15.38±2.64/  14.15±2.04 | 48 weeks | 2 times a week | ① |
| Lin^[89]^ | 2021 | China | CT/TR | 66.7±5.3/ 69.5±2.4 | 15/17 | 5.3±0.6/  4.2±1.1 | 8 weeks | 5 times a week | ①② |
| Song^[90]^ | 2020 | China | BDJ/TR | 67.9±3.5/68.2±3.3 | 68/52 | 2.9±0.5/  2.7±0.3 | 8 weeks | 5 times a week | ①③ |
| Qin^[91]^ | 2019 | China | VR/TR | 65.9±4.9/66.1±6.2 | 45/34 | 7.9±2.7/  8.1±2.3 | 6 weeks | 5 times a week | ①③ |
| He^[92]^ | 2019 | China | VRB/TR | 64.48±13.4/ 62.95±14.21 | 54/28 | NA | 4 weeks | 5 days a week | ① |
| Zhu^[93]^ | 2011 | China | TC/WE | 63.35±8.72/ 64.83±9.29 | 23/17 | 2.72±1.95/  2.78±2.29 | 4 weeks | 2 times a week | ①③ |
| Ji^[94]^ | 2016 | China | TC/TR | 56.06±11.16/ 59.13±11.22 | 17/15 | 2.09±1.07/  2.28±1.18 | 12 weeks | NA | 1. ③ |
| Wang^[95]^ | 2017 | China | AQE/TR | 63.93±6.95/ 64.45±6.82 | 38/22 | 4.05±1.55/  4.30±1.66 | 8 weeks | 5 times a week | ①②③ |
| Han^[96]^ | 2016 | China | BT/TR | 58.12±5.25/ 56.08±4.80 | 33/27 | 1.72±0.74 /  1.80±0.71 | 4 weeks | 5 times a week | ① |
| Yu^[97]^ | 2015 | China | OE/TR | 63.5±8.2/ 65.2±7.4 | 52/29 | 3.9±1.4/  4.1±1.3 | 12 weeks | NA | ①② |
| Tang^[98]^ | 2017 | China | FE/TR | 60.58±8.41 | 20/20 | NA | NA | NA | ①② |
| Luo^[99]^ | 2023 | China | CT/TR | 68.22±4.07/ 68.81±4.33 | 45/31 | 5.35±1.01/  5.12±1.02 | 8 weeks | NA | ① |
| Liu^[100]^ | 2017 | China | RAGT/TR | 58.7±9.8/56.5±10.8 | 25/15 | 3.1±1.1/  3.3±0.8 | 10 weeks | 2 times a week | ①②③ |
| Kunkel^[101]^ | 2017 | UK | DT/TR | 71.3±7.7/ 69.7±6.0 | 25/26 | 4.7±3.5/  7.0±4.9 | 10 weeks | 2 times a week | ①② |
| saNtos^[102]^ | 2017 | Brazil | BT/RT | 67.0±7.9/68.5±6.5 | 18/8 | 5.6±4.2/  5.4±5.3 | 12 weeks | NA | ① |
| Arias^[103]^ | 2009 | Spain | BT/TR | 66.90±11.11/66.55±5.57 | 9/12 | NA | 12 weeks | 2 times a week | ① |
| Landers^[104]^ | 2015 | USA | BT/CON | 70.1±9.5/ 74.3±8.8 | 13/7 | NA | 4 weeks | 3 times a week | ① |
| Combs^[105]^ | 2013 | America | BE/TR | 66.5±28.0/68.0±31.0 | 21/10 | NA | 12 weeks | NA | ①② |
| Sedaghati^[106]^ | 2015 | Iran | BT/TR | 59.13±8.37/ 57.22±6.87 | 21/9 | 2.53±0.5/  2.6±0.5 | 10 weeks | 3 times a week | ①② |
| van l^[107]^ | 2014 | Netherlands | GT/BT | 68.8±6.39/68.8±9.68 | 20/23 | 9.0 (4.00, 13.25)/  8.8 (2.50, 11.50) | 5 weeks | 2 times a week | ① |
| GÖZ1^[108]^ | 2021 | Turkey | PE/RT/CON | 64.0/65.0/ 61.0 | 15/5 | 42.0 (3.0–98.0)/  27.0 (1.5–60.0)/  27.0 (12.0–120)M | 6 weeks | 2 times a week | ① |
| Lu^[109]^ | 2024 | China | CT/TR | 66.70±6.73/66.57±6.82 | 34/26 | 5.93±2.34/  5.23±2.39 | 4 weeks | Once a week | ①②③ |
| Kalyani^[110]^ | 2020 | Australia | DT/TR | 65.24±11.88/66.50±7.70 | 13/20 | 8.67±5.83/  8.89±6.30/  8.41±5.29 | 12 weeks | 2 times a week | ①② |
| Pérez^[111]^ | 2018 | Spain | TC/TR | 66.3±6.1 | 12/17 | NA | 11 weeks | 2 times a week | ② |
| Wan^[112]^ | 2021 | China | QG/TR | 64.95±7.83/67.03±7.47 | 19/21 | 3.63±1.52/  3.25±1.73 | 12 weeks | 4 times a week | ② |
| Kwok^[113]^ | 2019 | China | YG/TR | 63.7±8.2/63.5±9.3 | 65/73 | NA | 8 weeks | NA | ②③ |
| [Hassan](https://www.researchgate.net/profile/Hassan-Daneshmandi?_tp=eyJjb250ZXh0Ijp7ImZpcnN0UGFnZSI6InB1YmxpY2F0aW9uIiwicGFnZSI6InB1YmxpY2F0aW9uIn19)^[114]^ | 2017 | Iran | PE/TR | 57±6.24/58.31±7.37 | 18/12 | 7.27±3.80/  8.19±3.14 | 8 weeks | 3 times a week | ② |
| Wróblewska^[115]^ | 2019 | Poland | DE/VR | 72.1±7.5/67.6±6.6 | 17/23 | 5.2±1.1 /  6.0±1.2 | 12 weeks | 2 times a week | ② |
| Guo^[116]^ | 2018 | China | QG/DR | 65.15±3.74/63.95±6.68 | 16/23 | NA | 12 weeks | 2 times a week | ②③ |
| Schlenstedt^[117]^ | 2015 | Germany | RT/TR | 75.7±5.5/75.7±7.2 | NA | 10.1±6.0/  9.3±7.9 | 8 weeks | 2 times a week | ② |
| Kaut^[118]^ | 2016 | Germany | WBV/CON | 66.10±8.28/ 67.92±8.78 | 36/20 | 7.03±6.48/  6.96±5.15 | 8 days | NA | ②③ |
| Schlick^[119]^ | 2016 | Germany | CT/TT | 71.2±10.9/68.9±6.8 | 6/14 | 10.4±5.2/  9.1±3.1 | 5 weeks | 2-3 times a week | ②③ |
| Collett^[120]^ | 2017 | UK | CR/OE | 66±9/67±7 | 61/44 | 4.8±4.1/  5.3±4.1 | 24 weeks | 2 times a week | ②③ |
| LV^[121]^ | 2021 | China | QG/TR | 65.87±6.13/ 63.25±6.70 | 11/20 | 5.60±1.72/  6.13±1.96 | 12 weeks | 5 times a week | ②③ |
| Luan^[122]^ | 2020 | China | TC/CI | 64.08±3.95/ 63.46±4.33 | 13/13 | NA | 6 weeks | 3 times a week | ②③ |
| Shen^[123]^ | 2021 | China | FAE/SE | 66.93±3.36/ 68.66±4.33 | 21/10 | 6.26±4.49 | 12 weeks | 2 times a week | ②③ |
| Duncan^[124]^ | 2012 | USA | DE/CON | 69.3±1.9/69.0±1.5 | 30/22 | 5.8±1.1/  7.0±1.0 | 48 weeks | NA | ③ |
| Mollinedo^[125]^ | 2017 | Spain | FE/PE | 66.00±13.14/62.85±9.75 | NA | 2.08±0.49/  2.00±0.82 | 48 weeks | 2 times a week | ③ |
| Corcos^[126]^ | 2013 | America | RT/CT | 58.6±5.6/59.0±4.6 | 28/20 | 6.5±4.7/  6.5±4.1 | 96 weeks | 2 times a week | ③ |
| Ebersbach^[127]^ | 2007 | Germany | WBV/TR | 72.5±6.0/ 75.0±6.8 | 14/7 | 7.0±3.3/  7.5±2.7 | 3 weeks | 3 times a week | ③ |
| Carroll^[128]^ | 2017 | Ireland | AQE/TR | 69.5/74 | 12/6 | NA | 6 weeks | 2 times a week | ③ |
| Moon^[129]^ | 2020 | America | QG/CON | 66.4±8.1/65.9±5.4 | 10/7 | 4.25±2.1/  5.33±3.3 | 12 weeks | 2 times a week | ③ |
| Xie^[130]^ | 2014 | China | DE/TR | 56. 63±8. 16/57. 80±8. 14 | 56/46 | 3. 57±1. 08/  3. 40±1. 31 | 8 weeks | 3 times a week | ③ |
| Chen^[131]^ | 2014 | China | FE/TR | 70.16±6.34/ 71.43±6.92 | 48/47 | 4.45±1.30/  4.10±1.26 | 24 weeks | 3 times a week | ③ |
| Frazzitta ^[132]^ | 2013 | Italy | AE/CON | 67±5/65±4 | NA | 8±5/  8±2 | 4 weeks | 5 times a week | ③ |
| McKee^[133]^ | 2013 | America | DE/CON | 68.4±7.5/ 74.4±6.5 | 20/13 | 7.0±5.5/  7.2±4.9 | 4 weeks | NA | ③ |
| Choi^[134]^ | 2007 | Korea | TC/CON | 60.81±7.6/65.54±6.8 | NA | 5.2±2.7/  5.2±2.7 | 12 weeks | Once a week | ② |
| Liu^[135]^ | 2017 | China | QG/TR | 57.1±7.0 | 15/26 | 4.1±1.0 | 10 weeks | 5 times a week | ②③ |

[1] YUANYUANA C, YANGA Y, SIQUANB L, et al. Effect of Virtual Ｒeality Technology Combined with Ｒehabilitation Training on Balance Function of Patients with Parkinson's Disease Medical Ｒecapitulate. 2019;25(21):4325-4329.

[2] JING L, ZHI Y, RUISONG L. The effect of virtual reality training on balance function in patients with Parkinson’s disease. Chinese Journal of Rehabilitation Medicine. 2020;35(06):682-687.

[3] WENJUAN D, CHENGPAN L, MIN S. Study on the effect of G-EO gait-therapy system on balance function in patients with Parkinson’s disease. Chinese Journal of Rehabilitation Medicine. 2022;37(04):494-500.

[4] HAI-HAO C, WEN-YU S, XIAO-MING X, et al. Effects of Wuqinxi on Balance, Walking and Quality of Life for Patients with Parkinson's Disease. Chin J Rehabil Theory Pract. 2021;27(09):1087-1092.

[5] XIHONG G, XIAZHU T, JIANMIN L. Effect of Tai Chi training on walking ability and fear of falling of patients with Parkinson’s disease. CHINESE NUＲSING ＲESEAＲCH 2016;30(28):3514-3517.

[6] BIN S, XIANG Q, YAN S, et al. Effects of vestibular rehabilitation training combined with gait training on Webster score, BBS score and fall incidence in elderly patients with Parkinson's disease. Chinese Journal of Gerontology. 2022;42(03):614-617.

[7] MIN Z, RUI B, SHAN L, et al. EffectofbalancefunctionrehabilitationtrainingontheimprovementofgaitdisorderinParkinson sdisease. Chinese Journal of Practical Nervous Diseases. 2021;24(09):781-786.

[8] LANGJUAN T, GUI M, ZHENZHEN W, et al. Effect of resistance training on improving lower limb muscle strength and balance function in patients with Parkinson's disease. Chinese Journal of Gerontology. 2019;39(01):127-130.

[9] VOLPE D, GIANTIN MG, MANUELA P, et al. Water-based vs. non-water-based physiotherapy for rehabilitation of postural deformities in Parkinson's disease: a randomized controlled pilot study. Clin Rehabil. 2017;31(8):1107-1115.

[10] PICELLI A, MELOTTI C, ORIGANO F, et al. Does robotic gait training improve balance in Parkinson's disease? A randomized controlled trial. Parkinsonism Relat Disord. 2012;18(8):990-993.

[11] DE NATALE ER, PAULUS KS, AIELLO E, et al. Dance therapy improves motor and cognitive functions in patients with Parkinson's disease. NeuroRehabilitation. 2017;40(1):141-144.

[12] GUADARRAMA-MOLINA E, BARRóN-GáMEZ CE, ESTRADA-BELLMANN I, et al. Comparison of the effect of whole-body vibration therapy versus conventional therapy on functional balance of patients with Parkinson's disease: adding a mixed group. Acta Neurol Belg. 2021;121(3):721-728.

[13] GAO Q, LEUNG A, YANG Y, et al. Effects of Tai Chi on balance and fall prevention in Parkinson's disease: a randomized controlled trial. Clin Rehabil. 2014;28(8):748-753.

[14] ZHANG TY, HU Y, NIE ZY, et al. Effects of Tai Chi and Multimodal Exercise Training on Movement and Balance Function in Mild to Moderate Idiopathic Parkinson Disease. Am J Phys Med Rehabil. 2015;94(10 Suppl 1):921-929.

[15] HASHIMOTO H, TAKABATAKE S, MIYAGUCHI H, et al. Effects of dance on motor functions, cognitive functions, and mental symptoms of Parkinson's disease: a quasi-randomized pilot trial. Complement Ther Med. 2015;23(2):210-219.

[16] LEE NY, LEE DK, SONG HS. Effect of virtual reality dance exercise on the balance, activities of daily living, and depressive disorder status of Parkinson's disease patients. J Phys Ther Sci. 2015;27(1):145-147.

[17] XIAO CM, ZHUANG YC. Effect of health Baduanjin Qigong for mild to moderate Parkinson's disease. Geriatr Gerontol Int. 2016;16(8):911-919.

[18] FENG H, LI C, LIU J, et al. Virtual Reality Rehabilitation Versus Conventional Physical Therapy for Improving Balance and Gait in Parkinson's Disease Patients: A Randomized Controlled Trial. Med Sci Monit. 2019;25:4186-4192.

[19] CAKIT BD, SARACOGLU M, GENC H, et al. The effects of incremental speed-dependent treadmill training on postural instability and fear of falling in Parkinson's disease. Clin Rehabil. 2007;21(8):698-705.

[20] PICELLI A, MELOTTI C, ORIGANO F, et al. Robot-assisted gait training is not superior to balance training for improving postural instability in patients with mild to moderate Parkinson's disease: a single-blind randomized controlled trial. Clin Rehabil. 2015;29(4):339-347.

[21] CLERICI I, MAESTRI R, BONETTI F, et al. Land Plus Aquatic Therapy Versus Land-Based Rehabilitation Alone for the Treatment of Freezing of Gait in Parkinson Disease: A Randomized Controlled Trial. Phys Ther. 2019;99(5):591-600.

[22] PALAMARA G, GOTTI F, MAESTRI R, et al. Land Plus Aquatic Therapy Versus Land-Based Rehabilitation Alone for the Treatment of Balance Dysfunction in Parkinson Disease: A Randomized Controlled Study With 6-Month Follow-Up. Arch Phys Med Rehabil. 2017;98(6):1077-1085.

[23] SMANIA N, CORATO E, TINAZZI M, et al. Effect of balance training on postural instability in patients with idiopathic Parkinson's disease. Neurorehabil Neural Repair. 2010;24(9):826-834.

[24] XIAO C, ZHUANG Y, KANG Y. Effect of Health Qigong Baduanjin on Fall Prevention in Individuals with Parkinson's Disease. J Am Geriatr Soc. 2016;64(11):e227-e228.

[25] CARVALHO A, BARBIRATO D, ARAUJO N, et al. Comparison of strength training, aerobic training, and additional physical therapy as supplementary treatments for Parkinson's disease: pilot study. Clin Interv Aging. 2015;10:183-191.

[26] VIEIRA DE MORAES FILHO A, CHAVES SN, MARTINS WR, et al. Progressive Resistance Training Improves Bradykinesia, Motor Symptoms and Functional Performance in Patients with Parkinson's Disease. Clin Interv Aging. 2020;15:87-95.

[27] MICHELS K, DUBAZ O, HORNTHAL E, et al. "Dance Therapy" as a psychotherapeutic movement intervention in Parkinson's disease. Complement Ther Med. 2018;40:248-252.

[28] ÇOBAN F, BELGEN KAYGıSıZ B, SELCUK F. Effect of clinical Pilates training on balance and postural control in patients with Parkinson's disease: a randomized controlled trial. J Comp Eff Res. 2021;10(18):1373-1383.

[29] KHUZEMA A, BRAMMATHA A, ARUL SELVAN V. Effect of home-based Tai Chi, Yoga or conventional balance exercise on functional balance and mobility among persons with idiopathic Parkinson's disease: An experimental study. Hong Kong Physiother J. 2020;40(1):39-49.

[30] POMPEU JE, MENDES FA, SILVA KG, et al. Effect of Nintendo Wii™-based motor and cognitive training on activities of daily living in patients with Parkinson's disease: a randomised clinical trial. Physiotherapy. 2012;98(3):196-204.

[31] RIBAS CG, ALVES DA SILVA L, CORRêA MR, et al. Effectiveness of exergaming in improving functional balance, fatigue and quality of life in Parkinson's disease: A pilot randomized controlled trial. Parkinsonism Relat Disord. 2017;38:13-18.

[32] SHIH MC, WANG RY, CHENG SJ, et al. Effects of a balance-based exergaming intervention using the Kinect sensor on posture stability in individuals with Parkinson's disease: a single-blinded randomized controlled trial. J Neuroeng Rehabil. 2016;13(1):78.

[33] CUGUSI L, SOLLA P, SERPE R, et al. Effects of a Nordic Walking program on motor and non-motor symptoms, functional performance and body composition in patients with Parkinson's disease. NeuroRehabilitation. 2015;37(2):245-254.

[34] KURT EE, BüYüKTURAN B, BüYüKTURAN Ö, et al. Effects of Ai Chi on balance, quality of life, functional mobility, and motor impairment in patients with Parkinson's disease<sup/>. Disabil Rehabil. 2018;40(7):791-797.

[35] BANG DH, SHIN WS. Effects of an intensive Nordic walking intervention on the balance function and walking ability of individuals with Parkinson's disease: a randomized controlled pilot trial. Aging Clin Exp Res. 2017;29(5):993-999.

[36] HACKNEY ME, EARHART GM. Effects of dance on movement control in Parkinson's disease: a comparison of Argentine tango and American ballroom. J Rehabil Med. 2009;41(6):475-481.

[37] SOLLA P, CUGUSI L, BERTOLI M, et al. Sardinian Folk Dance for Individuals with Parkinson's Disease: A Randomized Controlled Pilot Trial. J Altern Complement Med. 2019;25(3):305-316.

[38] LEE HJ, KIM SY, CHAE Y, et al. Turo (Qi Dance) Program for Parkinson's Disease Patients: Randomized, Assessor Blind, Waiting-List Control, Partial Crossover Study. Explore (NY). 2018;14(3):216-223.

[39] CHERUP NP, STRAND KL, LUCCHI L, et al. Yoga Meditation Enhances Proprioception and Balance in Individuals Diagnosed With Parkinson's Disease. Percept Mot Skills. 2021;128(1):304-323.

[40] TILLMANN AC, SWAROWSKY A, CORRêA CL, et al. Feasibility of a Brazilian samba protocol for patients with Parkinson's disease: a clinical non-randomized study. Arq Neuropsiquiatr. 2020;78(1):13-20.

[41] KASHIF M, AHMAD A, BANDPEI MAM, et al. Combined effects of virtual reality techniques and motor imagery on balance, motor function and activities of daily living in patients with Parkinson's disease: a randomized controlled trial. BMC Geriatr. 2022;22(1):381.

[42] PAZZAGLIA C, IMBIMBO I, TRANCHITA E, et al. Comparison of virtual reality rehabilitation and conventional rehabilitation in Parkinson's disease: a randomised controlled trial. Physiotherapy. 2020;106:36-42.

[43] SILVA AZD, ISRAEL VL. Effects of dual-task aquatic exercises on functional mobility, balance and gait of individuals with Parkinson's disease: A randomized clinical trial with a 3-month follow-up. Complement Ther Med. 2019;42:119-124.

[44] KASHIF M, ALBALWI AA, ZULFIQAR A, et al. Effects of virtual reality versus motor imagery versus routine physical therapy in patients with parkinson's disease: a randomized controlled trial. BMC Geriatr. 2024;24(1):229.

[45] VOLPE D, SIGNORINI M, MARCHETTO A, et al. A comparison of Irish set dancing and exercises for people with Parkinson's disease: a phase II feasibility study. BMC Geriatr. 2013;13:54.

[46] CHEN J, CHIEN HF, FRANCATO DCV, et al. Effects of resistance training on postural control in Parkinson's disease: a randomized controlled trial. Arq Neuropsiquiatr. 2021;79(6):511-520.

[47] VENTURA MI, BARNES DE, ROSS JM, et al. A pilot study to evaluate multi-dimensional effects of dance for people with Parkinson's disease. Contemp Clin Trials. 2016;51:50-55.

[48] ASHBURN A, FAZAKARLEY L, BALLINGER C, et al. A randomised controlled trial of a home based exercise programme to reduce the risk of falling among people with Parkinson's disease. J Neurol Neurosurg Psychiatry. 2007;78(7):678-684.

[49] YANG WC, WANG HK, WU RM, et al. Home-based virtual reality balance training and conventional balance training in Parkinson's disease: A randomized controlled trial. J Formos Med Assoc. 2016;115(9):734-743.

[50] PICELLI A, MELOTTI C, ORIGANO F, et al. Robot-assisted gait training versus equal intensity treadmill training in patients with mild to moderate Parkinson's disease: a randomized controlled trial. Parkinsonism Relat Disord. 2013;19(6):605-610.

[51] GANDOLFI M, GEROIN C, DIMITROVA E, et al. Virtual Reality Telerehabilitation for Postural Instability in Parkinson's Disease: A Multicenter, Single-Blind, Randomized, Controlled Trial. Biomed Res Int. 2017;2017:7962826.

[52] CARPINELLA I, CATTANEO D, BONORA G, et al. Wearable Sensor-Based Biofeedback Training for Balance and Gait in Parkinson Disease: A Pilot Randomized Controlled Trial. Arch Phys Med Rehabil. 2017;98(4):622-630.e623.

[53] ZHANGLU, ZHAOWOWA, GEYING. Additional power training in patients with Parkinson’s disease: a randomized controlled trial. Chinese Journal of Rehabilitation. 2024;39(06):344-349.

[54] HONG W, FUCHAO L, JING D, et al. Effect of dual task training on limb motor function in patients with early and middle stage Parkinson's disease. Chinese Journal of Health Medicine. 2024;26(01):107-109.

[55] VOLPE D, GIANTIN MG, MAESTRI R, et al. Comparing the effects of hydrotherapy and land-based therapy on balance in patients with Parkinson's disease: a randomized controlled pilot study. Clin Rehabil. 2014;28(12):1210-1217.

[56] QUTUBUDDIN A, REIS T, ALRAMADHANI R, et al. Parkinson's disease and forced exercise: a preliminary study. Rehabil Res Pract. 2013;2013:375267.

[57] ZHI-CHENG L, A-ZHEN C, YI-JING J, et al. Effects of Virtual Reality Balance Game on Balance Function for Parkinson's Disease. ChinJ Rehabil Theory Pract. 2016;22(09):1059-1063.

[58] LICHUN S, RONG C. Effects of virtual reality balance games combined with muscle strength training on balancefunction and motor ability of Parkinson's patients. Journal o f Hainan Medical University. 2020;26(09):655-658+663.

[59] CHENSI, JIE L, SHUN L, et al. Effects of Virtual Reality Rehabilitation on Balance for Patients with Parkinson's Disease. ChinJRehabil Theory Pract. 2017;23(09):1091-1095.

[60] YAN W. Study on the Intervention of Tango Dance in Patients with Parkinson's disease 2022.

[61] SUN G. Research on the Effects of Tai Chi on Motor Symptoms of Patients with Parkinson’s Disease and Its Promotion Based on the Internet Platform in Song Gao. 2022.

[62] ZHIGUO L, YUE M, YULINA, et al. Whole body vibration training combined with limb linkage training for patients with Parkinson's disease

Observation of motor function curative effect. Chin J Convalescent Med. 2021;30(01):75-77.

[63] DUCHUN Z, LIANG T, TONGCAI T, et al. Effects of whole body vibration combined with multiple motor strategy training on motor function and daily living ability of patients with Parkinson's disease. Chinese Journal of Rehabilitation Medicine. 2020;35(12):1486-1488.

[64] TONG Z, JIUYING S, CAIHUA S. Application of Ｒesistance Exercise Combined with Ｒegular Ｒehabilitation Training in the Nursing of Patients with Parkinson' s Disease. Qilu Nursing Journal. 2022;28(05):22-25.

[65] YI-MING W, GUANG-HUA L, XIAO-QIONG W, et al. Effect of progressive squat resistance training on motor function of patients with Parkinson disease. Chinese Journal of Clinical Medicine. 2023;30(03):520-524.

[66] BINGKUN C. Study on the effect of simple diabolo rehabilitation exercise on motor ability and depressive psychology of patients with mild Parkinson's disease. 2022.

[67] RUNZE L. An Application Study of Evidence-Based Virtual Reality Rehabilitation Training for Patients with Parkinson's Disease. 2021.

[68] FENGCHUN L, QIAN M, XIAOMENG S, et al. The application of Baduanjin exercise in patients with Parkinson's disease based on the theory of interactive standard. CHINESE NURSING RESEARCH. 2023;37(18):3388-3391.

[69] YING Z, HUI Q, YAJUN Z, et al. The effects of metoba combined with balance function training on balance and walking ability of patients with Parkinson's disease were studied based on gait analysis. Chinese Journal of Gerontology. 2022;42(14):3478-3480.

[70] BO L, HONG Y, DAN W. Influence of Taiji exercise on balance ability and fear of falling of patients with Parkinson's disease. Chinese Journal of Rehabilitation Medicine. 2017;32(03):309-312.

[71] FULIN L. Effect of Taijiquan on quality of life in elderly patients with Parkinson's disease. Chinese Journal of Gerontology. 2017;37(20):5121-5123.

[72] GUOJUAN Z, HONGXIA Z, JUAN L, et al. Application of Wuqinxi combined with physical training in patients with Parkinson's disease. Chinese Clinical Nursing. 2022;14(05):287-290.

[73] WEN H, TING D, XINYUAN D, et al. Application of motion-sensing games combined with five-fowl play in patients with motor disorders of Parkinson's disease. GuangxiMedical Journal. 2021;43(09):1153-1156+1160.

[74] LI D, JIANLAN C, XIAOMING Y, et al. Effect of Tai Chi Exercise on Mental Health and Balance Ability in Patients with Parkinson's Disease Shanghai Nursing. 2023;23(03):19-21.

[75] YE Y, JUNHUI S. Effect of Tai Chi balance exercise group therapy on improving balance function and depression in patients with Parkinson's disease. Guizhou Medical Journal. 2020;44(07):1071-1072.

[76] WEI S. Application effect of dual task training in patients with Parkinson's motor dysfunction. Modern Nurse. 2022;29(12):124-128.

[77] CHANGGUI Y, AIDONG L, SIMIN C. The effect of balance training system on balance disorder and fall of Parkinson＇s disease. Journal ofClinicalandExperimentalMedicine. 2018;17(24):2646-2649.

[78] QING Z, YING G, ZHIQIONG W, et al. Study on the intervention of Parkinson's health exercise on motor function of patients with Parkinson's disease. Journal of Nursing（China）. 2020;27(04):52-56.

[79] LANGJUAN T, YUYAN P, CHUNXIA W, et al. Effects of parkinsonian exercises and slow walking on motor function and balance function of limbs in patients with parkinsonian disease. Chinese Journal of Gerontology. 2020;40(13):2801-2803.

[80] GAI Z. Research on the Intervention Effect of Health Qigong Wuqinxi on theWalking and Balance Ability of Patients with Parkinson's Disease. 2019.

[81] AIJUN P, YINPING X, ZHAOYING P. Effects of body weight support treadmill training on cardiopulmonary endurance, balance ability and quality of life of elderly patients with Parkinson’s disease. Shanghai Pharmaceutical. 2023;44(06):51-54.

[82] YANMING Z, GAOYAN L, SONG WEIQUN, et al. Clinical observation of visual feedback-based postural control training to improve balance function and activities of daily living in patients with Parkinson's disease. Chinese Journal of Rehabilitation Medicine. 2023;38(06):775-779.

[83] XIAOMING X, BIHONGYAN. Effect of abdominaltorsion movement on depression,constipation,motorsymptomsand quality of life in patient swith Parkinson's disease. ChinJ Rehabil Theory Pract. 2022;28(02):220-226.

[84] MEIHUA W, MIN G, HAIQIN W, et al. Rehabilitation effect of Taijiquan with different training loads on patients with early and middle Parkinson's disease. Progress in Biochemistry and Biophysics. 2023;50(10):2487-2495.

[85] RAN W, HUIZI W, QINGJUAN W, et al. Intervention eff ect of bench exercise on gait, balance ability and quality of life of patients with Parkinson dyskinesia. Nursing Practice and Research. 2022;19(01):66-69.

[86] XINWEI S, WEILI Y, JUN J. Effects of Baduanjin combined with balance pad training on lower limb motor function and body balance in elderly patients with Parkinson's disease. Practical clinical integration of traditional Chinese and Western medicine. 2021;21(11):56-57.

[87] XIA C, XIANRONG M, MING Y, et al. Effect of XPH-B balance instrument combined with trunk core control on balance in patients with Parkinson＇s disease. Chinese geriatric health medicine. 2023;21(05):51-54.

[88] QIAN Z, FENG C, ZIJUAN J, et al. Application effect of Switch motion-sensing game combined with Wuqin Play in functional training of patients with Parkinson's disease. Modern Nurse. 2023;30(10):134-137.

[89] ZAILONG L, XIONGWEI F, CHAOWEI Y, et al. Effect of Flexbot lower extremity rehabilitation robot combined with virtual reality training on balance function and walking ability of patients with Parkinson's disease. Zhejiang medical science. 2021;43(04):405-408+413.

[90] ZHENGXIN S, ZENGLIN C, MIN W, et al. The value of Baduanjin combined with balance mat training in improving balance function in elderly patients with Parkinson's disease. Chinese Journal of Practical Nursing. 2020;36(2):100-104.

[91] LINGZHI Q, WEI L, XIAOJUAN W, et al. Application of virtual reality technology in frozen gait rehabilitation of Parkinson's disease. Chinese Journal of Physical Medicine and Rehabilitation. 2019;41(3):206-209.

[92] RONG H, LEI J. Intervention effect of virtual reality balance game combined with progressive rehabilitation nursing in patients with Parkinson's disease. Life science instrument. 2022;20(z1):192-193.

[93] YI Z, JIAN-XING L, NING L. Effect of Taijiquan on Motion Control for Parkinson＇s Disease at Early Stage. Chin J Rehabil Theory Pract. 2011;17(04):355-358.

[94] SUGIONG J, ZHIJUAN M, QINGMEI Y. Effectiveness of Tai Chi for Parkinson disease. Chinese Journal of Rehabilitation. 2016;31(01):51-53.

[95] YI⁃ZHAO W, HUA Z, SHI⁃CHUN F, et al. Effect of water⁃based exercise onmotor function, balance function andwalking ability in patients with Parkinson's disease. ChinJ Contemp Neurol Neurosurg. 2017;17(05):346-351.

[96] TINGTING H, HONG Y, MIN Z. Effeet of visual feedback balance training on the balance ability of early Parkinson's disease patients Chinese Journal of Rehabilitation. 2016;31(04):258-260.

[97] MEI Y, LIANTAO L, TONGBAO D, et al. Effect of strengthening core muscle strength training on rehabilitation of Parkinson's disease. Guangdong medicine. 2015;36(01):77-79.

[98] LANGJUAN T, YUYAN P, LICHUN Y. Observation of the therapeutic effect of Parkinson's health exercises on the rehabilitation of motor symptoms in patients with Parkinson's disease. Chinese Journal of Rehabilitation Medicine. 2017;32(04):464-466.

[99] FENG L. Effect of exercise combined with treadmill exercise training on motor function of patients with Parkinson's disease. Jilin Medical College. 2023;44(06):1652-1654.

[100] PING L, MEIYUN C. Eficacy of Lokomat roboticassisted gait training on improving gait function in patients with Parkinson's disease Chinese Journal of Rehabilitation. 2017;32(01):30-32.

[101] KUNKEL D, FITTON C, ROBERTS L, et al. A randomized controlled feasibility trial exploring partnered ballroom dancing for people with Parkinson's disease. Clin Rehabil. 2017;31(10):1340-1350.

[102] SANTOS SM, DA SILVA RA, TERRA MB, et al. Balance versus resistance training on postural control in patients with Parkinson's disease: a randomized controlled trial. Eur J Phys Rehabil Med. 2017;53(2):173-183.

[103] ARIAS P, CHOUZA M, VIVAS J, et al. Effect of whole body vibration in Parkinson's disease: a controlled study. Mov Disord. 2009;24(6):891-898.

[104] LANDERS MR, HATLEVIG RM, DAVIS AD, et al. Does attentional focus during balance training in people with Parkinson's disease affect outcome? A randomised controlled clinical trial. Clin Rehabil. 2016;30(1):53-63.

[105] COMBS SA, DIEHL MD, CHRZASTOWSKI C, et al. Community-based group exercise for persons with Parkinson disease: a randomized controlled trial. NeuroRehabilitation. 2013;32(1):117-124.

[106] SEDAGHATI P, DANESHMANDI H, KARIMI N, et al. A Selective Corrective Exercise to Decrease Falling and Improve Functional Balance in Idiopathic Parkinson's Disease. Trauma Mon. 2016;21(1):e23573.

[107] VAN DEN HEUVEL MR, KWAKKEL G, BEEK PJ, et al. Effects of augmented visual feedback during balance training in Parkinson's disease: a pilot randomized clinical trial. Parkinsonism Relat Disord. 2014;20(12):1352-1358.

[108] GöZ E, ÇOLAKOĞLU BD, ÇAKMUR R, et al. Effects of Pilates and Elastic Taping on Balance and Postural Control in Early Stage Parkinson's Disease Patients: A Pilot Randomised Controlled Trial. Noro Psikiyatr Ars. 2021;58(4):308-313.

[109] CHUNXIA L, YOUZHEN Z, XIANRONG M, et al. Clinical effect of Baduanjin exercise combined with G-EO rehabilitation robot in treatment of dyskinesia due to Parkinson’s disease:An analysis of 30 cases. HUNAN JOURNAL OF TRADITIONAL CHINESE MEDICINE. 2024;40(04):18-21.

[110] KALYANI HH, SULLIVAN KA, MOYLE GM, et al. Dance improves symptoms, functional mobility and fine manual dexterity in people with Parkinson disease: a quasi-experimental controlled efficacy study. Eur J Phys Rehabil Med. 2020;56(5):563-574.

[111] PéREZ-DE LA CRUZ S. A bicentric controlled study on the effects of aquatic Ai Chi in Parkinson disease. Complement Ther Med. 2018;36:147-153.

[112] WAN Z, LIU X, YANG H, et al. Effects of Health Qigong Exercises on Physical Function on Patients with Parkinson's Disease. J Multidiscip Healthc. 2021;14:941-950.

[113] KWOK JYY, KWAN JCY, AUYEUNG M, et al. Effects of Mindfulness Yoga vs Stretching and Resistance Training Exercises on Anxiety and Depression for People With Parkinson Disease: A Randomized Clinical Trial. JAMA Neurol. 2019;76(7):755-763.

[114] HASANDANESHMANDI, SHAGHAYEGHSAYYAR, BABAKBAKHSHAYESH. Theeffectof aSelectivePilates programonFunctionalBalanceand Falling Risk in Patients with Parkinson’s Disease. zahedan JRes Med sci. 2017:e7886.

[115] WRóBLEWSKA A, GAJOS A, SMYCZYŃSKA U, et al. The Therapeutic Effect of Nordic Walking on Freezing of Gait in Parkinson's Disease: A Pilot Study. Parkinsons Dis. 2019;2019:3846279.

[116] YADONG G. Effects of Health Qigong on Motor Function in Patientswith mild to Moderate Parkinson's Disease. 2018.

[117] SCHLENSTEDT C, PASCHEN S, KRUSE A, et al. Resistance versus Balance Training to Improve Postural Control in Parkinson's Disease: A Randomized Rater Blinded Controlled Study. PLoS One. 2015;10(10):e0140584.

[118] KAUT O, BRENIG D, MAREK M, et al. Postural Stability in Parkinson's Disease Patients Is Improved after Stochastic Resonance Therapy. Parkinsons Dis. 2016;2016:7948721.

[119] SCHLICK C, ERNST A, BöTZEL K, et al. Visual cues combined with treadmill training to improve gait performance in Parkinson's disease: a pilot randomized controlled trial. Clin Rehabil. 2016;30(5):463-471.

[120] COLLETT J, FRANSSEN M, MEANEY A, et al. Phase II randomised controlled trial of a 6-month self-managed community exercise programme for people with Parkinson's disease. J Neurol Neurosurg Psychiatry. 2017;88(3):204-211.

[121] CHUANFANG L. Effects of Health Qigong exercise on lower limb motor functionin patients with Parkinson's disease. 2021.

[122] XIN L. Effects of Innovative Tai Chi Training on Motor Ability and Quality of Life in Patients with Early Parkinson’s Disease. 2020.

[123] MENGYUE S. Cognition of Five-Animal Frolics Exercise on Patients with Mild and Moderate Parkinson's Disease and Motor Function Intervention Effect Research. 2021.

[124] DUNCAN RP, EARHART GM. Randomized controlled trial of community-based dancing to modify disease progression in Parkinson disease. Neurorehabil Neural Repair. 2012;26(2):132-143.

[125] MOLLINEDO-CARDALDA I, CANCELA-CARRAL JM, VILA-SUáREZ MH. Effect of a Mat Pilates Program with TheraBand on Dynamic Balance in Patients with Parkinson's Disease: Feasibility Study and Randomized Controlled Trial. Rejuvenation Res. 2018;21(5):423-430.

[126] CORCOS DM, ROBICHAUD JA, DAVID FJ, et al. A two-year randomized controlled trial of progressive resistance exercise for Parkinson's disease. Mov Disord. 2013;28(9):1230-1240.

[127] EBERSBACH G, EDLER D, KAUFHOLD O, et al. Whole body vibration versus conventional physiotherapy to improve balance and gait in Parkinson's disease. Arch Phys Med Rehabil. 2008;89(3):399-403.

[128] CARROLL LM, VOLPE D, MORRIS ME, et al. Aquatic Exercise Therapy for People With Parkinson Disease: A Randomized Controlled Trial. Arch Phys Med Rehabil. 2017;98(4):631-638.

[129] MOON S, SARMENTO CVM, STEINBACHER M, et al. Can Qigong improve non-motor symptoms in people with Parkinson's disease - A pilot randomized controlled trial? Complement Ther Clin Pract. 2020;39:101169.

[130] DONGQIN X, LING Y, LANYUN Y. Effects of dance training combined with cognitive intervention on cognitive function, motor function and coping style of patients with Parkinson's disease and prognosis analysis. Journal of Navy Medicine. 2024;45(03):323-326.

[131] DEFANG C, WEI C, GUIYUN W, et al. Application of Health Exercises and Cognitive Task Training in Parkinson's Patients With Cognitive Impairment. CHINA HEALTH STANDARD MANAGEMENT. 2024;15(09):176-180.

[132] FRAZZITTA G, MAESTRI R, GHILARDI MF, et al. Intensive rehabilitation increases BDNF serum levels in parkinsonian patients: a randomized study. Neurorehabil Neural Repair. 2014;28(2):163-168.

[133] MCKEE KE, HACKNEY ME. The effects of adapted tango on spatial cognition and disease severity in Parkinson's disease. J Mot Behav. 2013;45(6):519-529.

[134] CHOI HJ. Effects of therapeutic Tai chi on functional fitness and activities of daily living in patients with Parkinson disease. J Exerc Rehabil. 2016;12(5):499-503.

[135] XIAOLEI L, ZHIRONG W, MENGQING S, et al. Effects of Health Qigong on treatments of Parkinson’s disease. Chin J Neuroimmunol &, Neurol. 2017;24(01):34-37.
