# Supplementary figures and images for: Which exercise intervention is most promising for Parkinson's balance? A network meta-analysis
Source: Front Aging Neurosci. 2026 Jul 15;18:1879017. doi: 10.3389/fnagi.2026.1879017 (PMC13416450; doi:10.3389/fnagi.2026.1879017)

## SUCRA Rankings for BBS

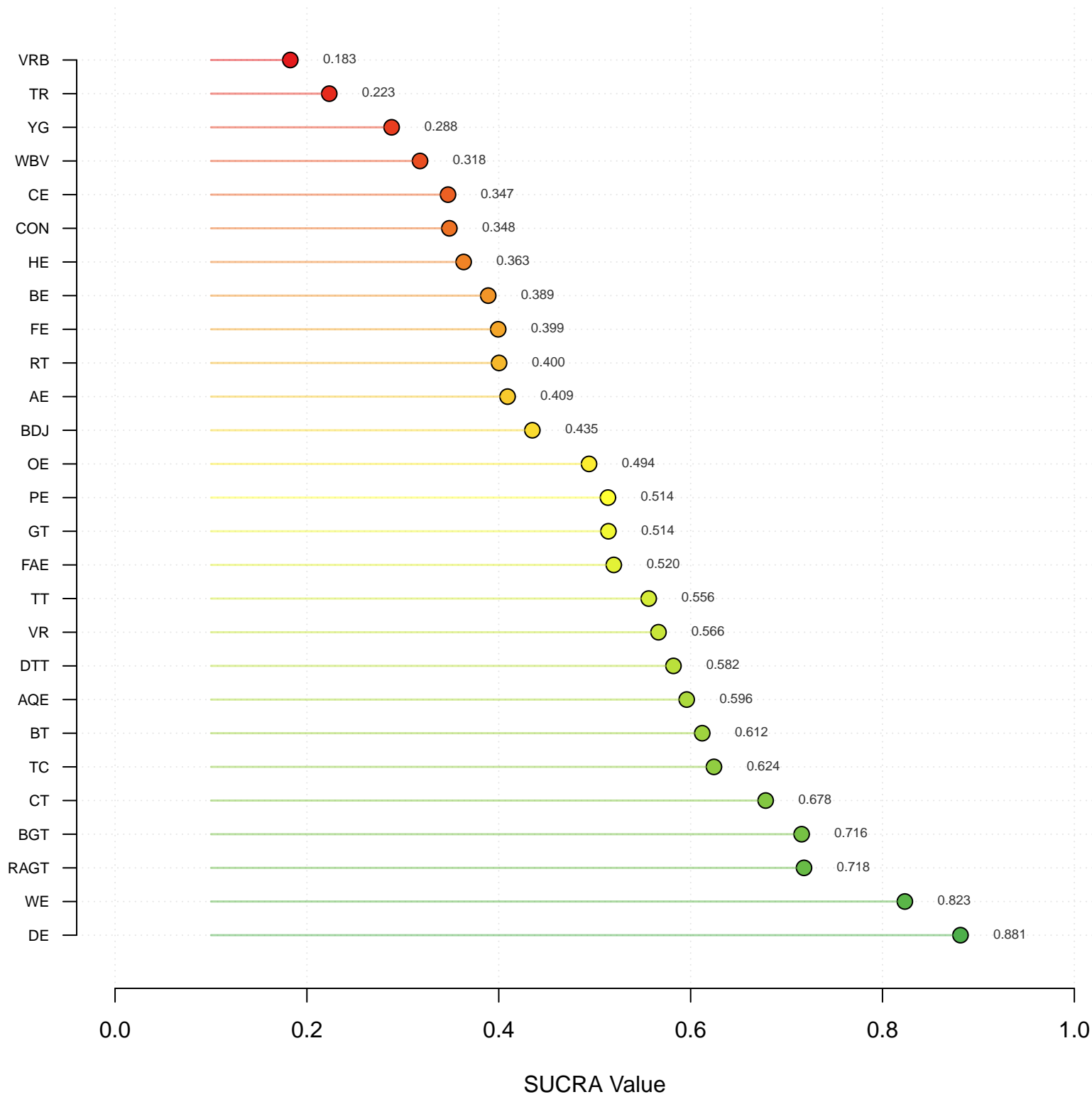

Supplement: Supplementary file 1 [file Data_Sheet_1.zip › Supplementary materials/Appendix 6.1-BBS SUCRA.pdf]

## SUCRA Rankings for TUG

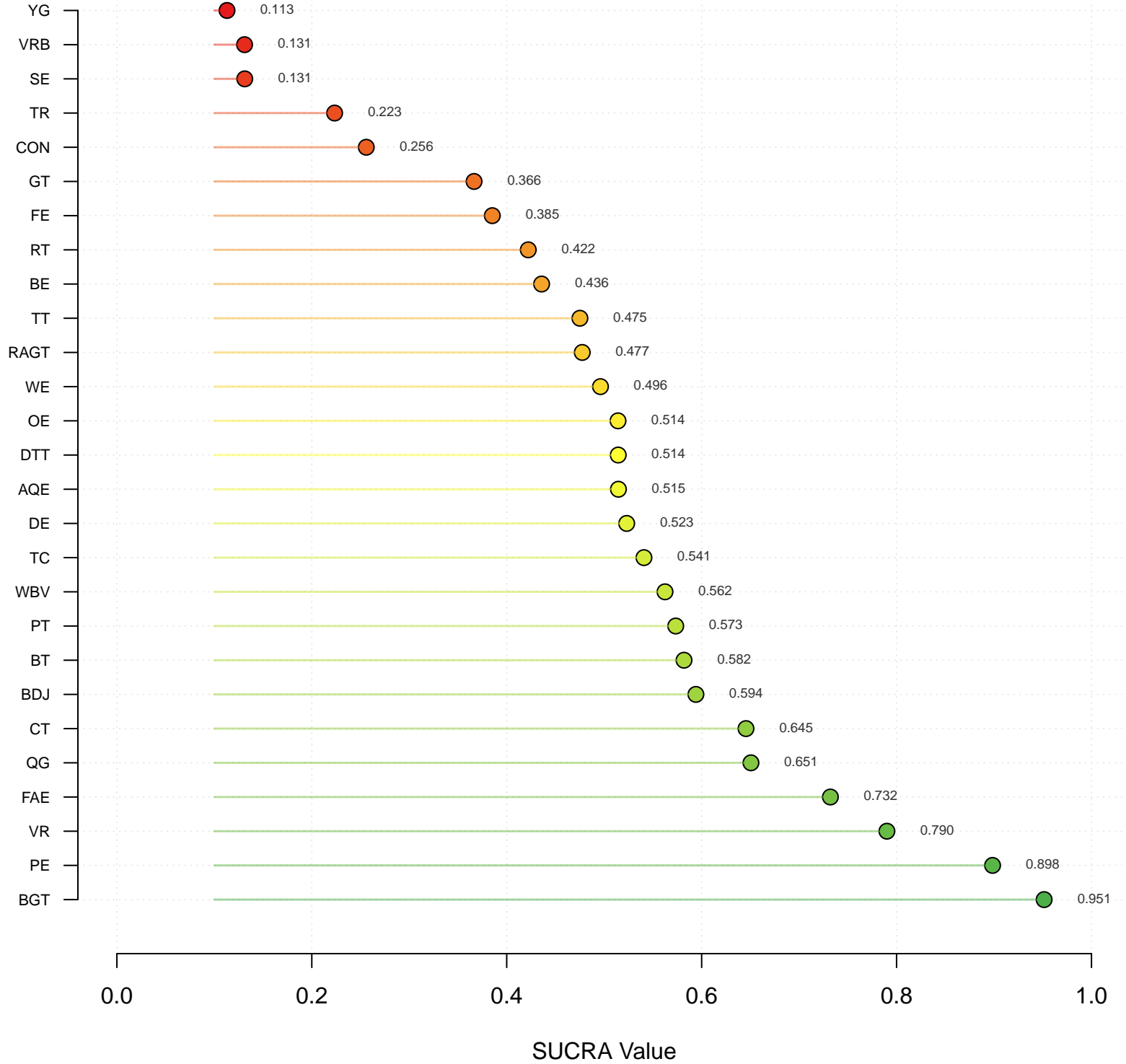

Supplement: Supplementary file 1 [file Data_Sheet_1.zip › Supplementary materials/Appendix 6.2-TUG SUCRA.pdf]

## SUCRA Rankings for UPDRS

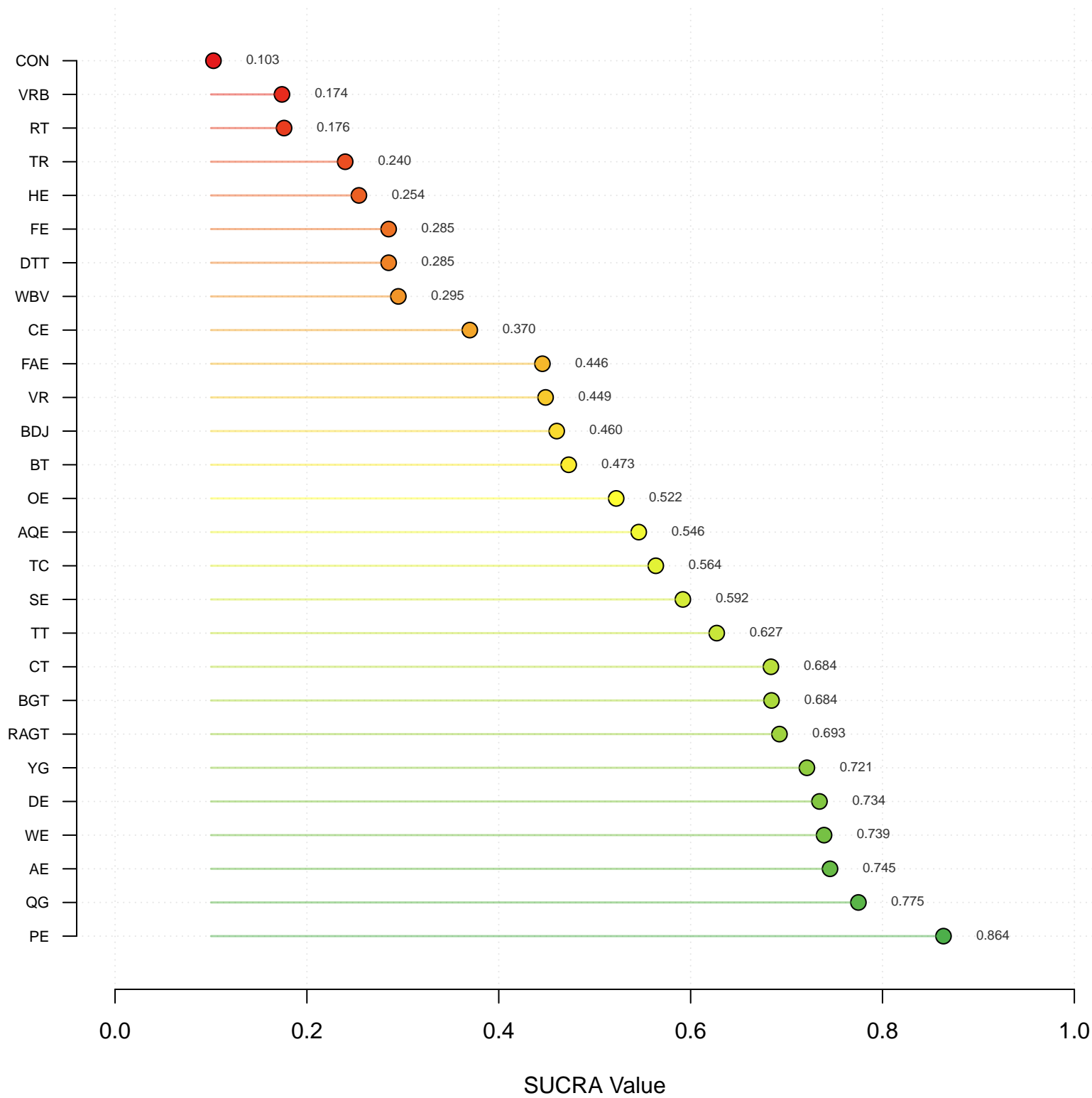

Supplement: Supplementary file 1 [file Data_Sheet_1.zip › Supplementary materials/Appendix 6.3-UPDRS-III SUCRA.pdf]

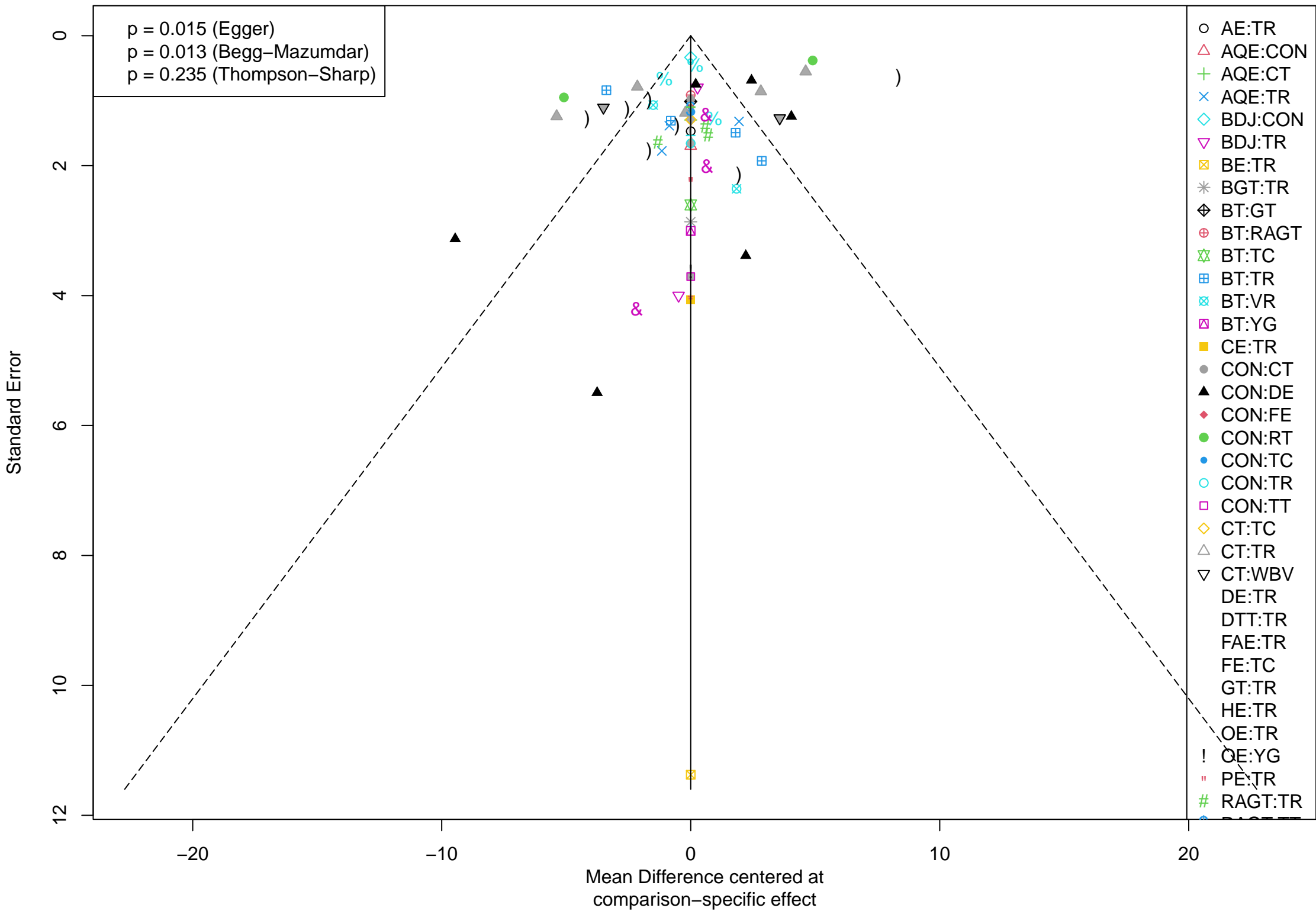

Supplement: Supplementary file 1 [file Data_Sheet_1.zip › Supplementary materials/Appendix 7.1-Funnel polt.pdf]

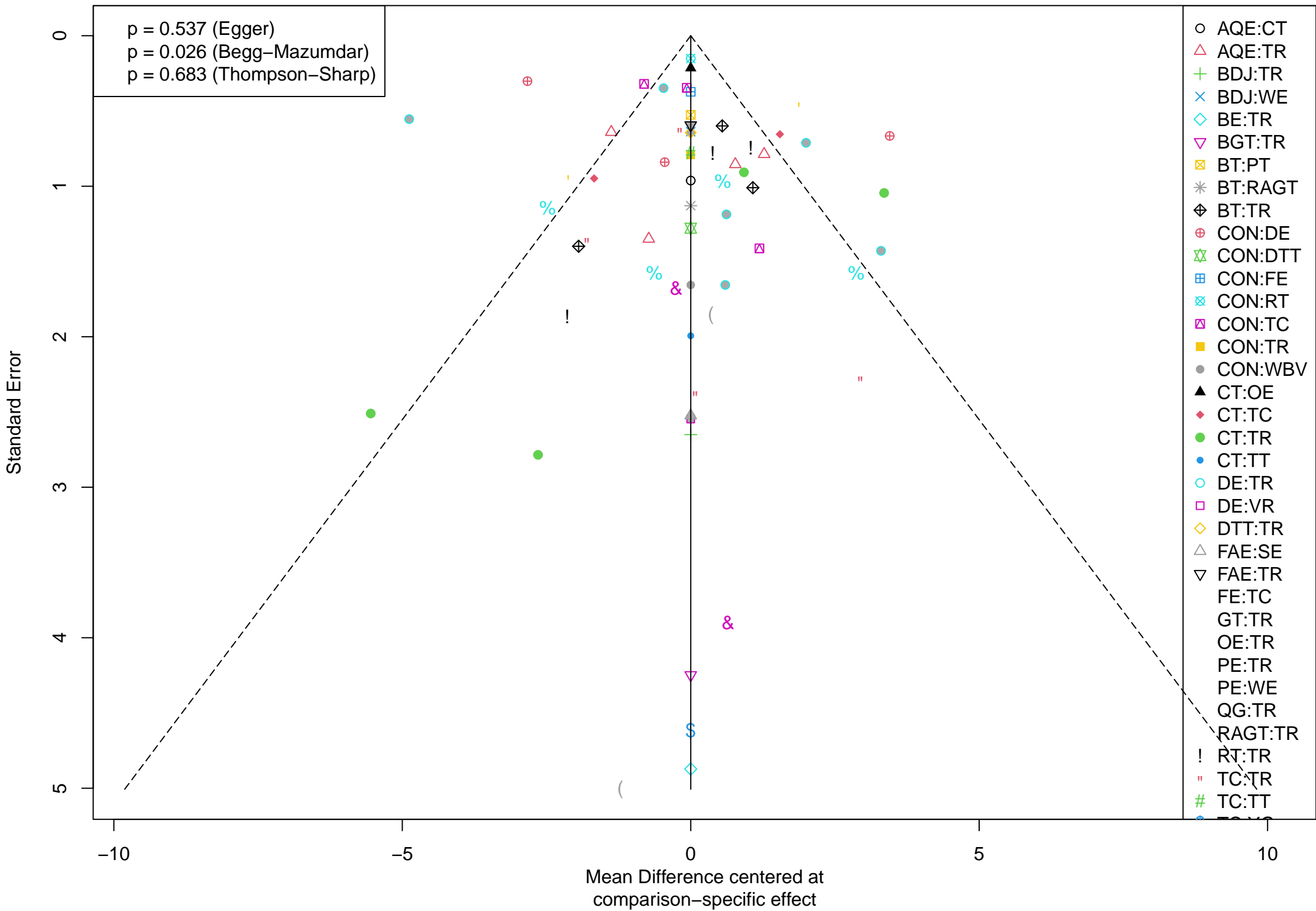

Supplement: Supplementary file 1 [file Data_Sheet_1.zip › Supplementary materials/Appendix 7.2-Funnel polt.pdf]

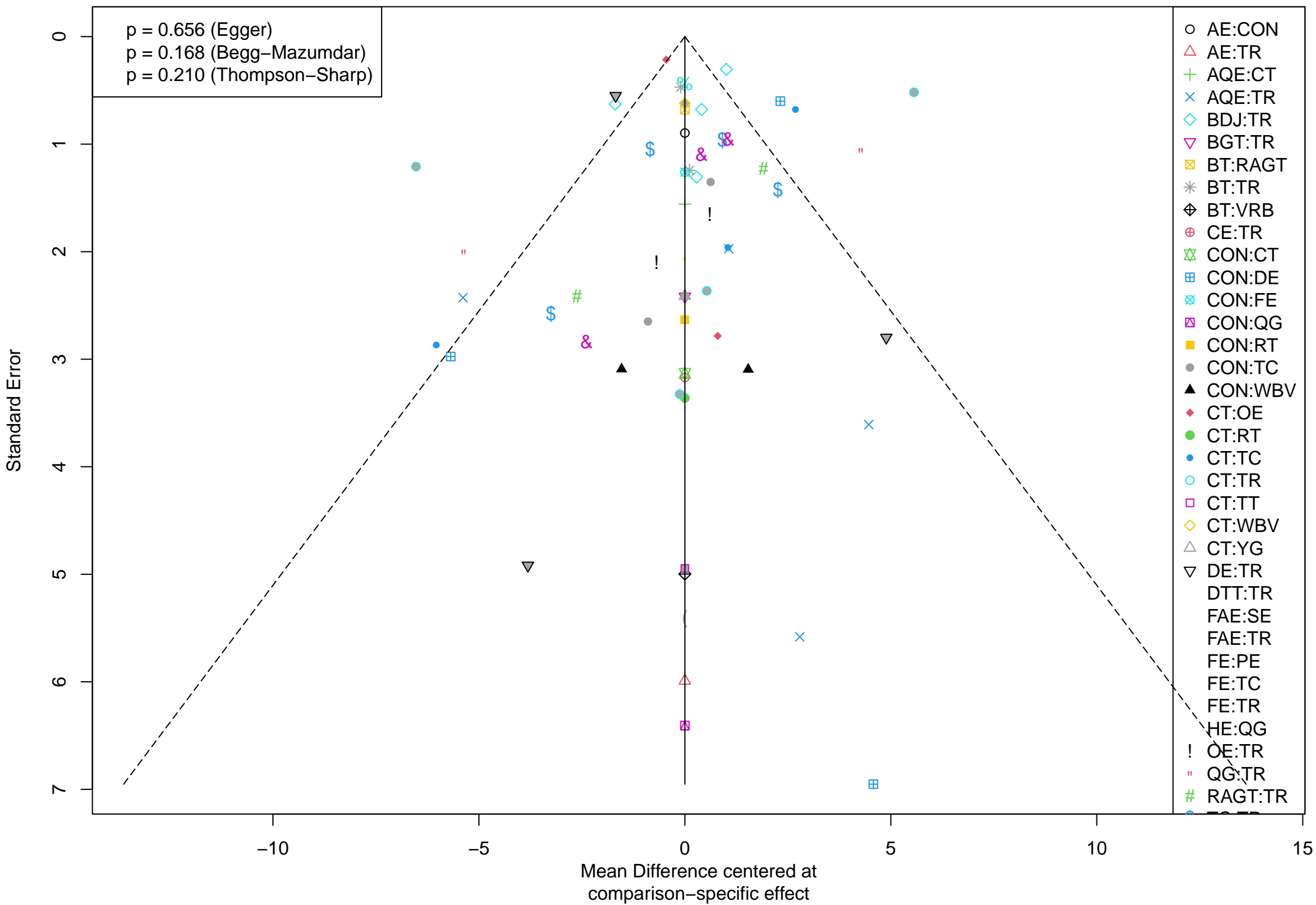

Supplement: Supplementary file 1 [file Data_Sheet_1.zip › Supplementary materials/Appendix 7.3-Funnel polt.pdf]

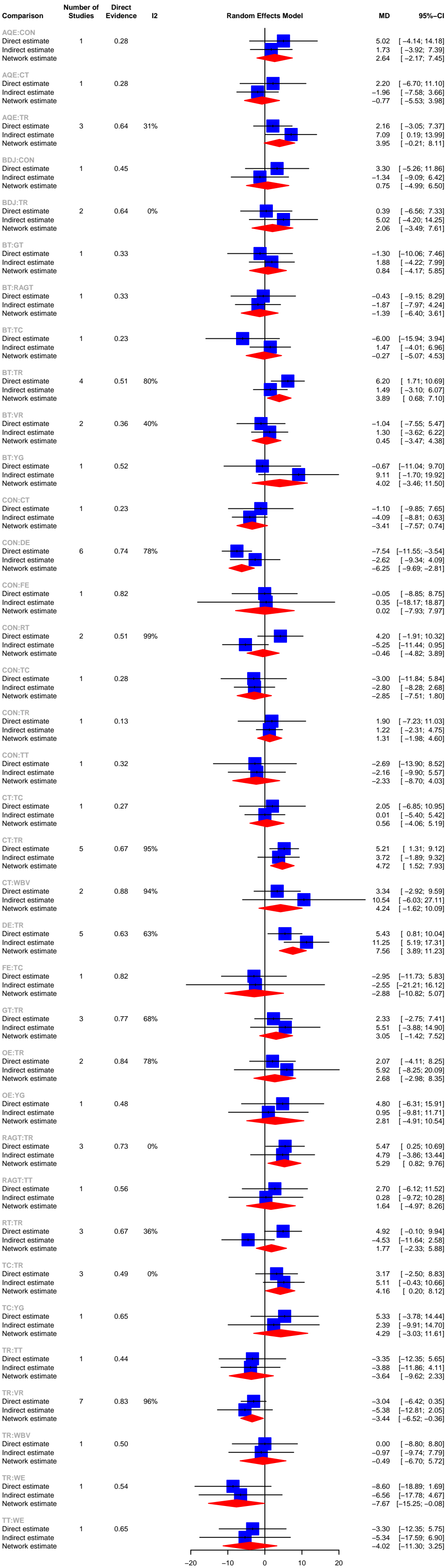

Supplement: Supplementary file 1 [file Data_Sheet_1.zip › Supplementary materials/Appendix 9.1-Node split.pdf]

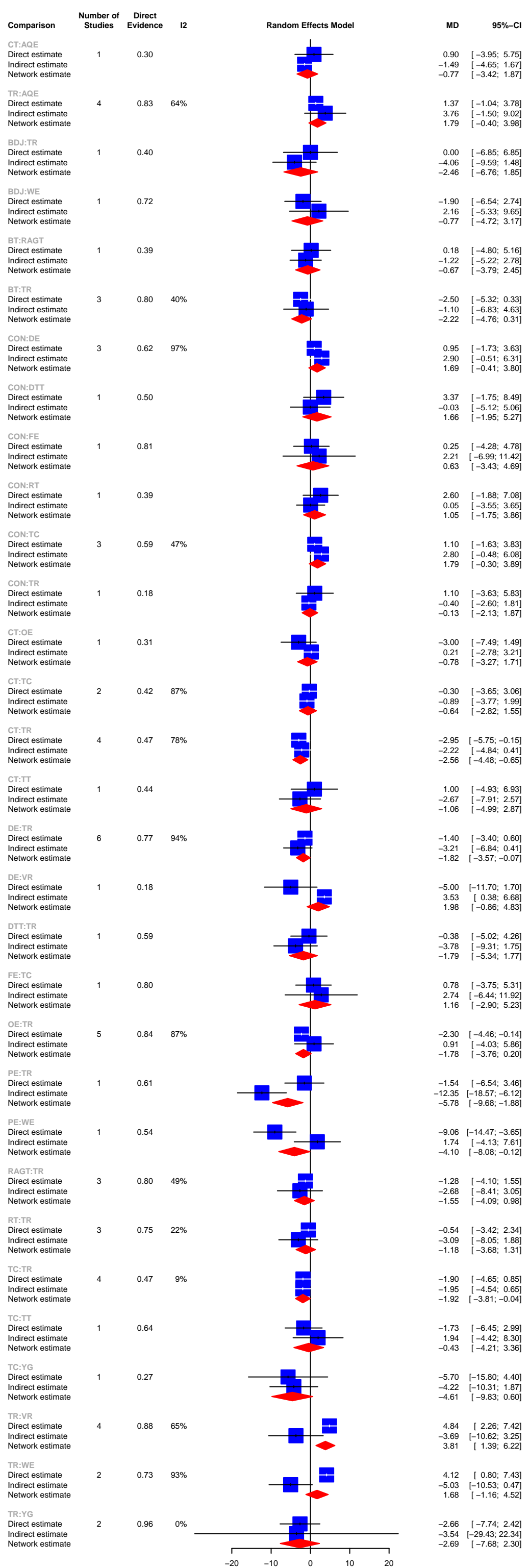

Supplement: Supplementary file 1 [file Data_Sheet_1.zip › Supplementary materials/Appendix 9.2-Node split.pdf]

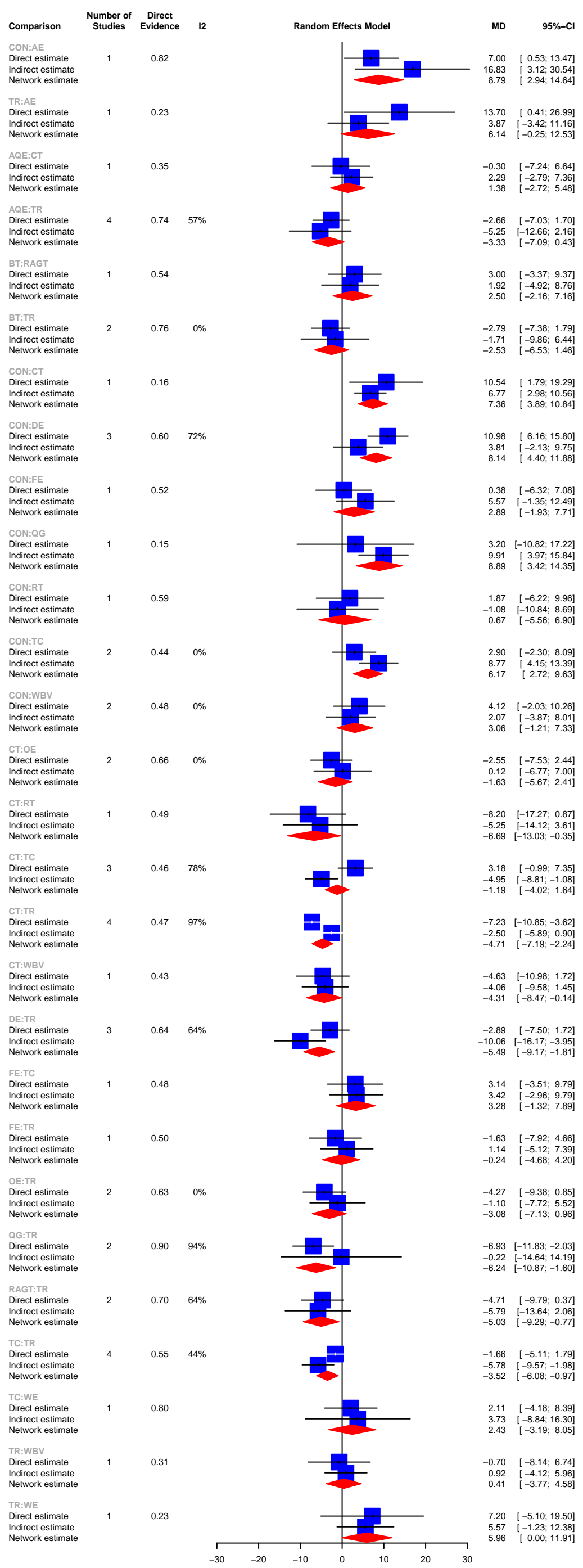

Supplement: Supplementary file 1 [file Data_Sheet_1.zip › Supplementary materials/Appendix 9.3-Node split.pdf]
